# Nectar Replaced by Volatile Secretion: A Potential New Role for Nectarless Flowers in a Bee-Pollinated Plant Species

**DOI:** 10.3389/fpls.2018.01243

**Published:** 2018-09-05

**Authors:** Elza Guimarães, Priscila Tunes, Luiz D. de Almeida Junior, Luiz C. Di Stasi, Stefan Dötterl, Silvia R. Machado

**Affiliations:** ^1^Laboratory of Ecology and Evolution of Plant-Animal Interactions, Department of Botany, Institute of Biosciences, São Paulo State University, Botucatu, Brazil; ^2^Graduation Program in Biological Sciences, Laboratory of Ecology and Evolution of Plant-Animal Interactions, Department of Botany, Institute of Biosciences, São Paulo State University, Botucatu, Brazil; ^3^Laboratory of Phytomedicine, Pharmacology and Biotechnology, Department of Pharmacology, São Paulo State University, Botucatu, Brazil; ^4^Department of Biosciences, University of Salzburg, Salzburg, Austria; ^5^Laboratory of Research in Plant Anatomy and Ultrastructure, Department of Botany and Centre of Electron Microscopy, Institute of Biosciences, São Paulo State University, Botucatu, Brazil

**Keywords:** nectar secretion, nectariferous and nectarless flowers, nectary anatomy and ultrastructure, plant–pollinator interactions, volatile compound secretion

## Abstract

The presence of nectarless flowers in nectariferous plants is a widespread phenomenon in angiosperms. However, the frequency and distribution of nectarless flowers in natural populations, and the transition from nectariferous to nectarless flowers are poorly known. Variation in nectar production may affect mutualism stability, since energetic resource availability influences pollinators’ foraging behavior. Here, we described the spatial and temporal nectar production patterns of *Jacaranda oxyphylla*, a bee-pollinated species that naturally presents nectarless flowers. Additionally, we compared nectariferous and nectarless floral disks in order to identify histological, subcellular and chemical changes that accompanied the loss of nectar production ability. For that we used standard methods for light and transmission electron microscopy, and gas chromatography coupled to mass spectrometry for chemical analyses. We verified that 47% of flowers did not produce nectar during the whole flower lifespan (nectarless flowers). We also observed remarkable inter-plant variation, with individuals having only nectarless flowers, others only nectariferous ones and most of them showing different proportions of both flower types, with variable nectar volumes (3–21 μl). Additionally, among nectariferous flowers, we registered two distinct rhythms of nectar production. ‘Early’ flowers produced nectar from 0 to 24 h, and ‘late’ flowers produced nectar from 24 to 48 h of anthesis. Although disks from nectariferous and nectarless flowers displayed similar histological organization, they differed strongly at subcellular level. Nectariferous (‘early’ and ‘late’) flowers exhibited a cellular apparatus typical of nectar secretion, while nectarless flowers exhibited osmophoric features. We found three aliphatic and one aromatic compound(s) that were detected in both the headspace of flowers and the disks of nectarless flowers, but not the disks of nectariferous flowers Although the remarkable variation in nectar availability may discourage pollinator visits, nectarless flowers might compensate it by producing volatile compounds that can be part of floral scent, acting as chemical attractants. Thus, nectarless flowers may be helping to maintain pollination in this scenario of trophic resource supply scarcity. We suggest that *J. oxyphylla* can be transitioning from a nectar-based pollination system to another resource-based or even to a deceit mechanism of pollination.

## Introduction

The characteristics of floral attractants, including primary ones, such as trophic resources, and secondary ones, such as chemical and visual signals, have strong influence on the establishment of plant–animal interactions (Chittka and Thomson, 2001; Armbruster and Muchhala, 2009; Schaefer and Ruxton, 2011). Floral nectar appeared on the late Cretaceous (Labandeira, 2002) and has since become key trophic resource mediating plant–pollinator interactions (Willmer, 2011). However, spatial and temporal variation in nectar production is commonly described in angiosperms, with differences reported among and within species, plants, and flowers (Pacini and Nepi, 2007; Lu et al., 2015; Zhao et al., 2016). Pollinators can react to variations in nectar features, and the optimal foraging theory, based on caloric consumption, has succeeded to explain their foraging behavior (Pyke, 2010, 2016). So, variation in nectar production may affect mutualism stability by influencing pollinators’ foraging behavior (Real, 1981), which may compromise plant reproductive fitness (Zhao et al., 2016). Thus, characterizing how this trophic resource is spatially distributed and how it is temporally released by flowers in a natural population could help to assess the impact of the presence of nectarless flowers on nectarivores’ visitation.

Bignoniaceae is known by the presence of zoophilous flowers (Gentry, 1974), with most species presenting nectar as trophic resource, which is produced by a conspicuous nectariferous annular disk that surrounds the ovary base (Galetto, 1995). However, some Bignoniaceae species may present nectarlessness flowers, which has been associated with the absence of a disk (Hauk, 1997), or with the presence of vestigial and non-secretory disks (Rivera, 2000) and with pollination by deceit (Umaña et al., 2011). In spite of *Jacaranda oxyphylla* Cham. being referred as a plant species that possesses a cylindrical nectary disk (Gentry and Morawetz, 1992), around half of its flowers was nectarless in natural populations (Guimarães et al., 2008). However, the causes and consequences of this phenomenon remain unknown. In other plant families, the transitions from nectarless plant species to nectariferous ones has been suggested to rely on subcellular modification, since no morphological differences between nectariferous and nectarless species have been found (Hobbhahn et al., 2013). However, nectary changes related to variation in nectar production within species remains unexplored. Thus, understanding the cellular basis driving the performance of nectariferous and nectarless flowers is essential to explain intra-species nectar variability. Here, we aimed to describe the spatial and temporal variation in nectar production at population level. Additionally, we performed a comparative investigation of the chemical composition and subcellular apparatus of nectariferous and nectarless floral disks in order to identify functional variations that might have accompanied the loss of nectar production ability. Finally, we discussed the potential ecological implications of presenting nectariferous and nectarless flowers focusing on plant–pollinator interactions.

## Materials and Methods

### Study Site and Plant Species

This study was conducted in natural populations of savanna physiognomies “Cerrado” located in Pratânia (22° 48′52″S, 48° 44′35″W) and Botucatu municipalities (22° 57′ 38″S, 48° 31′ 22″W) in São Paulo, Brazil. The field study was performed during the blooming period of the species that occurred at the end of dry season (August–October). This study is part of a long-term project that started in 2006 and is still active, and the data presented here has been collected in the years 2006, 2010–2011, and 2017.

**FIGURE 1:**
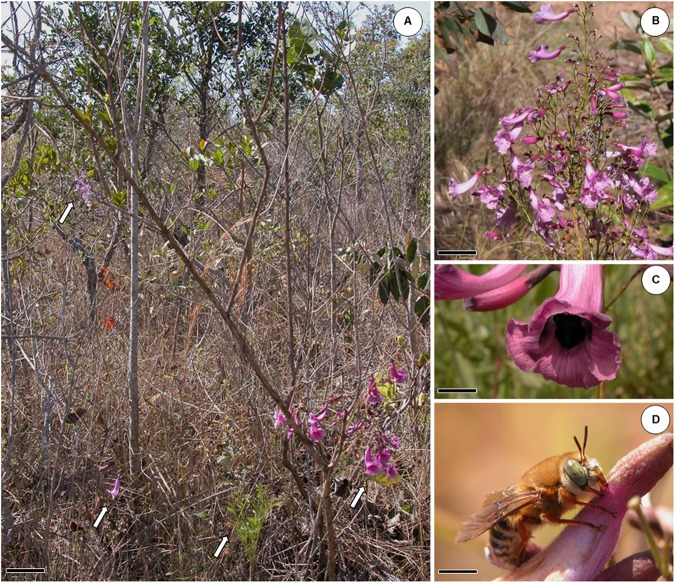
*Jacaranda oxyphylla* (Bignoniaceae) and its floral visitors. **(A)** Study site showing “Cerrado” vegetation and four individuals of *J. oxyphylla* (arrows). Scale bar: 13 cm; **(B)** detail of an inflorescence of *J. oxyphylla*. Scale bar: 5 cm; **(C)** *Eulaema nigrita*, one of the pollinators of *J. oxyphylla*, visiting a flower. Scale bar: 1.5 cm; **(D)** *Oxaea flavescens*, the main nectar robber of *J. oxyphylla* flowers, visiting a flower. Scale bar: 0.5 cm.

*Jacaranda oxyphylla* Cham. (Bignoniaceae) varies from sub-shrubby to shrubby habit (**Figure 1A**) and presents branchlets with bipinnate leaves, inflorescences as terminal panicles (**Figure 1B**) bearing flowers with cupular calyx, tubular–campanulate magenta to purplish blue corolla above a narrow basal tube (Gentry and Morawetz, 1992), which corresponds to the nectar chamber (Guimarães et al., 2008). Flowers present didynamous stamens with dithecate anther and a long sub-exerted staminode, a flattened–ovate ovary slightly contracted at the base to a cylindrical disk; elliptic thinly woody fruit with hyaline-membranaceous seeds (Gentry and Morawetz, 1992). Medium-sized bees *Eulaema nigrita* (**Figure 1C**) and *Bombus morio*, small-sized bee *Exomalopsis fulvofasciata* and, occasionally, hummingbirds visited the flowers in a legitimate way and behaved as pollinators; while *Oxaea flavescens* (**Figure 1D**) and *Xylocopa* sp. acted as nectar robbers (Guimarães et al., 2008).

Vouchers are deposited in the ‘Irina Delanova Gemtchujnicov’ Herbarium (BOTU) of the Biosciences Institute of the São Paulo State University (UNESP), Botucatu, Brazil, under numbers 24408–24412.

### Nectar Production Variability

For the nectar sampling described in subsections “Characterizing Nectar Production in Space: Variation Within and Among Plants” and “Characterizing Nectar Production in Time: Variation Throughout Anthesis,” we maintained all the sampled flowers isolated with bridal veil bags, since bud stage, in order to prevent nectar withdrawn by floral visitors, as recommended by Corbet (2003). The nectar volume was always measured using graded glass syringes (10 μl).

#### Characterizing Nectar Production in Space: Variation Within and Among Plants

In order to characterize floral nectar availability in space, considering both within and among plants variation in nectar production, we described the spatial distribution of *J. oxyphylla*, by measuring the Cartesian distances among plants in a natural population (15 plots of 100 m^2^, totaling 1,500 m^2^) and calculating Morisita’s dispersion index (Morisita, 1959, 1962). To characterize the variation in the amount of nectar potentially available to pollinators, we sampled all the 48 h and 72 h flowers in 29 plants (totaling 205 flowers, 7 ± 4 flowers per plant).

Then, we determined the percentage of nectarless flowers in our study population and we evaluated the variation of nectar production within plants by determining the proportion of nectariferous and nectarless flowers per plant. Additionally, we compared the frequency of nectariferous and nectarless flowers among plants using Pearson’s Chi-squared test, in order to verify if the plants showed distinct proportions of both types of flowers. Also, we evaluated if the mean volume of nectar produced by flowers varied among plants, which allowed us to identify if individual plants had an influence on nectar volume production. For this, we used ANOVA with Brown–Forsythe correction for heteroskedastic data and Games–Howell *post hoc* test for pairwise comparisons. Additionally, we counted the number of inflorescences per plant (*n* = 40 plants) and the number of flowers per inflorescence (*n* = 46 inflorescences, 40 plants). We verified if there was any influence of flower position within inflorescences on the accumulated nectar volume by using a regression analysis (*n* = 35 inflorescences, 18 plants). We also verified the probability of finding nectarless flowers in the apex and the base of inflorescences (*n* = 22 inflorescences, 14 plants), using generalized linear model (GLM) with Binomial error distribution.

#### Characterizing Nectar Production in Time: Variation Throughout Anthesis

We determined whether nectar accumulation started in bud stage by inspecting pre-anthesis floral buds (*n* = 30 buds from 20 plants). In order to verify if nectarless flowers have no nectar during their whole lifespan or if they were actually product of nectar resorption, in addition to characterize the nectar secretion pattern, we described the daily nectar secretion (with removal) and the accumulated nectar production throughout anthesis. To evaluate nectar secretion with removal during the whole flower lifespan, we sampled flowers at intervals of 24 h, starting at the moment of flower opening in the first day of anthesis (0 h) and ending at the seventh day of anthesis, totaling 144 h of monitoring. We finished our sampling at the seventh day because, at that moment, only 15% of the flowers were still attached to the inflorescences. For that, we used a set of 45 bagged flowers (*n* = 20 plants, 1–3 flowers per plant). Every 24 h, we removed each individual bag, withdrew all the nectar from each flower, and immediately bagged it again. We resampled the same flowers every 24 h until corolla abscission or until 144 h of anthesis. Then, in order to identify if the daily nectar production (with removal) differed among days of anthesis, we compared the volumes of nectar produced at each day, using ANOVA with Brown–Forsythe correction for heteroskedastic data and Games–Howell *post hoc* test for pairwise comparisons. We also performed Local Weighted Regression (LOESS) with 95% confidence intervals to describe the variation in daily nectar production throughout the anthesis. To determine the accumulated nectar volume, we performed a set of experiments in which we sampled the nectar in flowers at every 24 h from 0 h to 48 h of anthesis (*n* = 81 flowers from 39 plants, 1–3 flowers sampled per plant). However, instead of resampling the same flowers at every interval, we sampled the accumulated nectar volume in a different set of flowers each time. So that, each set of flowers was sampled just once and then discarded. Around 0700 h (time of flower opening), in the first day of anthesis, we sampled nectar from a set of 30 flowers (0-h flowers). In sequence, 28 different flowers were sampled at 0700 h in the second day of anthesis (24-h flowers), and 23 flowers in the third day of anthesis (48-h flowers). We also verified if the volume of accumulated nectar varied among days of anthesis using Kruskal–Wallis test, and performed LOESS with 95% confidence intervals to describe the secretion pattern in flowers with accumulated nectar. Additionally, we performed Wilcoxon Rank Sum test to verify if there were any differences between the sum of the daily nectar production (with removal) and the accumulated nectar (during the part of anthesis in which there was nectar production) in order to investigate if there was any effect of nectar removal on secretion pattern. The sum of the daily nectar production at 24 h corresponds to the volume withdrawn at 0 h + 24 h, and the sum of the daily nectar production at 48 h corresponds to the volume withdrawn at 0 h + 24 h + 48 h.

Thirty-eight flowers used in the accumulated nectar experiment were nectarless. The remaining nectariferous flowers (*n* = 43 from 30 plants, 1–3 flowers per plant) were used to determine total concentration of nectar (% w/w) along the three time intervals (0 h, 24 h, 48 h) by mean of a hand-held refractometer. We used both nectar volume and concentration parameters to estimate the total milligrams of sugar produced per flower, as proposed by Galetto and Bernardello (2005). Then, we compared nectar concentration and total milligrams of sugar (mgS) per flower throughout anthesis using ANOVA with Brown–Forsythe correction for heteroskedastic data and one-way ANOVA, respectively. All the statistical analyses were performed in R v. 3.3.1 (R Development Core Team, 2016) and in R v. 3.4.3 (R Development Core Team, 2018) with standard and additional packages: ggplot2 (Wickham, 2009), msir (Scrucca, 2011), and userfriendlyscience (Peters, 2017).

### Histological and Cellular Analyses

We performed Kruskal–Wallis rank sum test and we found that nectariferous and nectarless flowers did not differ in their longevity [X^2^_(1)_ = 0.0571, *p* = 0811]. We sampled flowers of both types based on the periods of nectar secretion of nectariferous flowers, from 0 until 48 h of anthesis. We also sampled disks after nectar production cessation (72 h of anthesis). We also compared the disk volume of nectariferous and nectarless flowers by measuring the height and the diameter with a digital caliper (Mytutoyo^®^, United States) in 13 flowers, from eight plants.

For histological characterization of the disks, we fixed disk samples (*n* = 10 for each flower type) in Karnovsky’s solution (4% paraformaldehyde; 1% glutaraldehyde in 0.1 M phosphate buffer, pH 7.2; 0.2 M phosphate buffer, pH 7.2) for 24 h (Karnovsky, 1965), and we dehydrated them in an ethanol series (50, 70, 90, 100%) and embedded them in methacrylate resin (Historesin^®^, Leica, Wetzlar, Germany) in accordance with the manufacturer’s recommended procedure. We obtained the sections (4–6 μm) using a Leica RM2255 rotary microtome and we stained them with 0.05% toluidine blue, pH 4.5 (O’Brien et al., 1964). We carried out histochemical tests on material fixed in Karnovsky solution, both in sections obtained by free hand and by microtome after inclusion in resin. We applied the following histochemical tests: 10% aqueous ferric chloride solution for phenolic compounds identification (Johansen, 1940); Lugol for the identification of starch (Johansen, 1940); Sudan IV for lipids in general (Johansen, 1940), Sudan Black B for lipids in raw nectar, as described by Kram et al. (2008); and NADI’s reagent (α-naphtol and *N*,*N*-dimethyl-*p*-phenylenediamine) for the detection of resin or essential oils (David and Carde, 1964). The presence of phenolic substances was checked by staining with toluidine blue, according to Ramalingam and Ravindranath (1970). We analyzed the slides under a Leica DMR microscope with image capture system (Leica DFC 425).

For ultrastructural analyses, we fixed disk fragments in glutaraldehyde (2.5% with 0.1 M phosphate buffer, pH 7.3, for 6–8 h at 4°C) and post-fixed them with osmium tetroxide (1% in the same buffer, for 2 h at room temperature). After a washing in distilled water, we stained the materials with 0.5% uranyl acetate in water solution for 2 h at room temperature. Afterward, we dehydrated the samples in a graded acetone series (50, 70, 90, and 100%), and embedded them in Araldite^®^ resin at room temperature. We carried out the polymerization at 60°C for 48 h and stained the semi-thin sections with 1% toluidine blue, while ultra-thin sections were stained with uranyl acetate and lead citrate (Reynolds, 1963). We observed the sections under transmission electron microscopy (TEM), Tecnai Spirit (FEI) microscope, at 80 kV.

### Chemical Analysis

To verify if the disks of both nectariferous and nectarless flowers contain volatiles that are also found in the headspace of flowers, we compared the volatile compounds present in the disks with the floral scent of *J. oxyphylla*.

To evaluate the chemical compounds present in the disks, we collected disks from 15 nectariferous and 18 nectarless flowers (*n* = 5 plants for both types of flowers), and separated them from the flowers using razor blades. All of the flowers were bagged since bud stage and were collected at approximately 48 h of anthesis. Disk samples were stored in a freezer at approximately -80°C. Later, we analyzed the samples on a Thermo Scientific GC-MS, model FOCUS equipped with an automatic sampler (Thermo – triplus DUO) and coupled to a Thermo – ISQ 230ST mass detector. We used a TG-5MS column for the analysis (30 m long, 0.25 mm of inner diameter, 0.25 μm of film thickness) and we maintained a constant 1 mL/min flow of helium as the carrier gas. The disks were accommodated in vials at 200°C for 15 min in a heating stove prior to the injection. An automated gas tight syringe was maintained at 140°C, perforated the vial seal, collected 2 mL of sample from inside the vial and immediately injected the sample into the GC injector in splitless mode, with the injector temperature being 200°C. The samples were co-injected with a 500 μL mixture of n-alkanes (C7–C30) at 0.1% of concentration that was added to the vials containing the disks. Column temperature was initially 50°C, then increased by 5°C/min to 250°C and kept constant for 5 min. The MS interface was at 250°C. The detector was operated in electron impact ionization mode (70 eV), with a scanning range of 34–350 m/z. Given that cutting and heating of the disks will produce compounds not normally released in the headspace of the flowers, we only looked for compounds detected in *in situ* samples of floral scents. To obtain such samples, we sampled floral scent by dynamic headspace, following the protocol by Dötterl et al. (2005). The sampled flowers (*n* = 5 flowers from five plants at 0 h of anthesis) were enclosed for 10 min in polyethylene bags (8 × 10 cm). As only recently opened flowers were used for the analyses, it was not possible to effectively determine in the field if these flowers were nectariferous or nectarless, as we found that some nectariferous flowers only start nectar accumulation at 24 h (‘late’ flowers, see Results section). The volatile compounds which accumulated inside the bags were collected with adsorbent traps connected to a membrane pump, with an air flow of 200 mL/min during 50 min. We used adsorbent tubes with approximately 15 mm of length by 2 mm of internal diameter that were filled with a mixture of 1.5 mg Tenax-TA (60–80 mesh) and 1.5 mg of Carbotrap B (20–40 mesh; both Supelco^®^). Besides collecting volatile compounds directly from the flowers, we also collected samples from leaves in order to discriminate any possible contaminants or not flower-exclusive volatile compounds. Samples were stored in a freezer at approximately -80°C. We analyzed the samples on an automated thermo desorption system (Model TD-20; Shimadzu, Kyoto, Japan) coupled to a GC-MS (model QP2010 Ultra EI; Shimadzu) equipped with a ZB-5 fused silica column (60 m long, 0.25 mm of inner diameter, 0.25 μm of film thickness), as described in Mitchell et al. (2015). We maintained a constant 1.5 mL/min flow of helium as the carrier gas. The injector temperature was 200°C. Oven temperature started at 40°C, then increased by 6°C/min to 250°C and kept constant for 1 min. The MS interface was at 250°C. Mass spectra were taken at electron energy 70 eV (in EI mode), with scanning range of 30–350 m/z.

In all of the analysis, we carried out tentative compound identification using NIST 08, and Adams (2007) mass spectral libraries. Final identification was carried out by comparing the mass spectra and Kovats Retention Indices (RI) of target compounds with that of authentic standard compounds. For quantitative analysis of VOCs, 100 ng each of ca. 150 components, among them monoterpenes, aliphatic, and aromatic compounds, were injected into the GC-MS system. The mean of the peak areas (total ion current) of these compounds was used to estimate the total amount of scent available in the scent samples (Etl et al., 2016).

## Results

### Nectar Production Variability

#### Characterizing Nectar Production in Space: Variation Within and Among Plants

**FIGURE 2:**
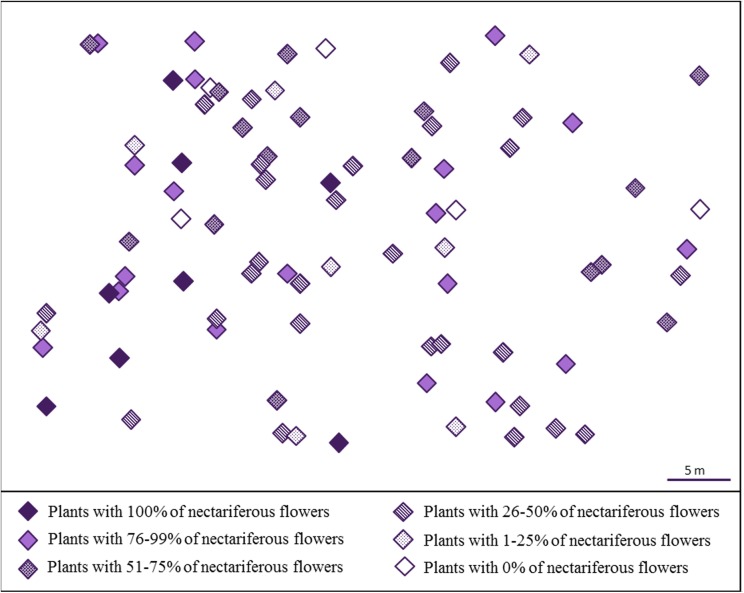
Spatial distribution of *J. oxyphylla* (Bignoniaceae), Botucatu, Brazil. Each diamond represents a single plant. The colors and patterns inside the diamonds indicate the percentage of nectariferous flowers found in each plant.

**FIGURE 3:**
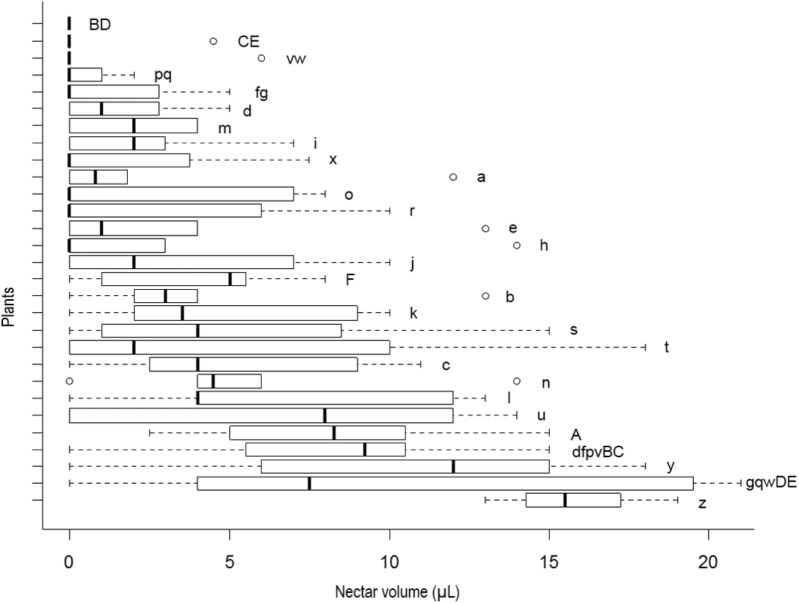
Box plots of nectar volume variability per plant in *J. oxyphylla*. The box plots show the median (vertical line across the box), 25th and 75th percentiles (lower and upper edges of the box) and the upper and lower whiskers, which correspond to the higher and lower data that is no further from the box than 1.5 times the interquartile range. Any data that lied beyond the whiskers was considered an outlier (empty circles). Nectar volumes significantly different from one plant to another are denoted by different letters on the right side of the boxes (ANOVA with Brown–Forsythe correction for heteroskedastic data and Games–Howell *post hoc* test). Nectar volume showed high variability within and among plants (*p* < 0.05). Note that various plants presented flowers with no nectar and others presented outliers as well.

We observed that *J. oxyphylla* presents a clumped spatial distribution (*I*_σ_ = 0.5013, 95% IC) (**Figure 2**). We noticed that only 12.5% of *J. oxyphylla* individuals presented more than one inflorescence (2–3), and the plants presented a mean of 6.1 ± 3.9 open flowers per inflorescences per day. We found 47% of nectarless flowers in the study population. The frequency of nectariferous and nectarless flowers differed among plants [X^2^_(30)_ = 94.49, *P* < 0.001], with 10% of plants showing only nectariferous flowers, 6.5% of plants showing only nectarless flowers and the remaining plants showing variable mixed proportions of both flower types (**Figure 2**). So, we observed a significant influence of plant on nectar volume production (*F*_28,55.98_ = 4.2, *P* < 0.001) (**Figure 3**). Also, nectar volume was quite variable among nectariferous flowers within each plant (3–21 μL) (**Figure 3**). There was no association between flowers position in the inflorescence and the accumulated nectar volume per flower (*R*^2^ = -0.0285, *P* = 0.9758). Additionally, the probability of finding nectarless flowers was similar when comparing the basis and the apex of the inflorescences (*Z* = 0.096, *P* = 0.924).

#### Characterizing Nectar Production in Time: Variation Throughout Anthesis

**FIGURE 4:**
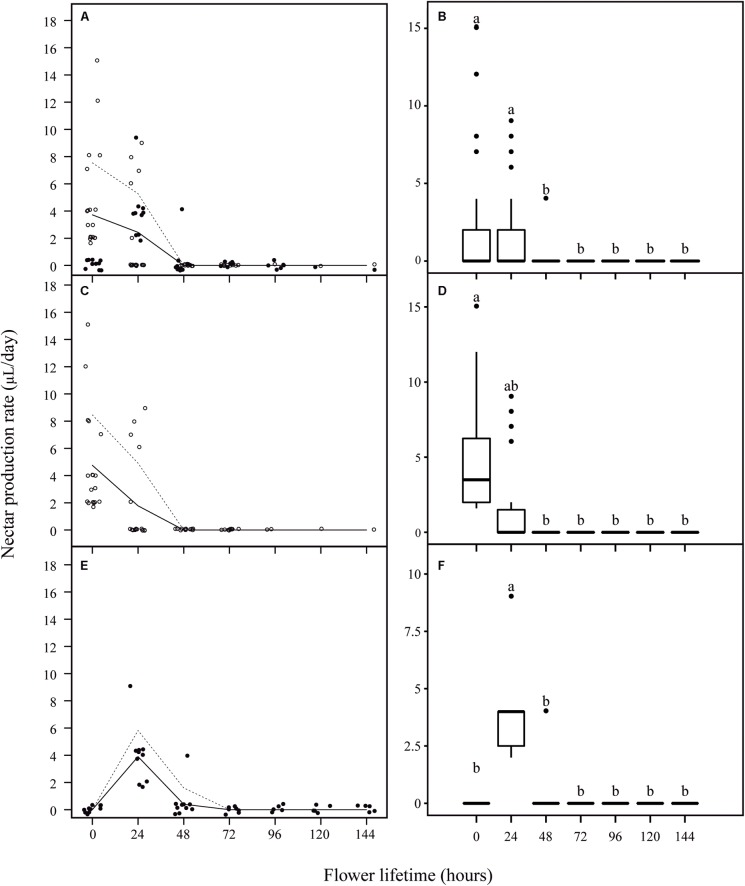
Nectar production rate per flower lifetime in *J. oxyphylla* flowers. In **(A,C,E)**, the trendlines describe a visual relationship between the two variables (nectar production rate and flower lifetime) based on the lowest smoother using a locally weighted regression (LOESS). Dashed lines are 95% confidence interval upper limits. The 95% confidence interval lower limits were zero and the line was omitted. The empty circles represent ‘early’ flowers and the full circles represent ‘late’ flowers. In **(B,D,F)**, the box plots show the median (horizontal line across the box), 25th and 75th percentiles (lower and upper edges of the box) and the upper and lower whiskers, which correspond to the higher and lower data that is no further from the box than 1.5 times the interquartile range. Any data that lied beyond the whiskers was considered an outlier (filled circles). Nectar production rates significantly different from one period to another are denoted by different letters above the boxes (ANOVA with Brown–Forsythe correction for heteroskedastic data and Games–Howell *post hoc* test). **(A)** Nectar production rate per flower at every 24 h of anthesis. **(B)** Nectar production rate was similar during the first 2 days of anthesis, followed by an undermost production during the third day of anthesis and ceasing before 72 h of anthesis (*p* < 0.05); **(C)** nectar production rate per ‘early’ flowers at every 24 h of anthesis; **(D)** in ‘early’ flowers, the maximum volume of nectar occurred at 0 h, followed by the addition of smaller amounts of nectar until 24 h and by production cessation (*p* < 0.05); **(E)** nectar production rate per ‘late’ flowers at every 24 h of anthesis; **(F)** in ‘late’ flowers, the maximum volume of nectar registered at 24 h, followed by a smaller production until 48 h and by production cessation (*p* < 0.05).

No nectar was found in pre-anthesis bud stage (1 day before anthesis), and nectar presence was registered only at the moment of flower opening or later. We observed more nectarless flowers in the first day of anthesis than in the following day (**Figure 4A**). Actually, the majority of these first-day nectarless flowers remained nectarless throughout their lifespan. However, part of the flowers that showed no nectar during their first day of anthesis, started producing nectar later on. Considering all the sampled nectariferous flowers, we observed a variation in the daily rate of nectar production per flower during anthesis (*F*_6,88.85_ = 9.03, *P* < 0.001) (**Figures 4A,B**), with a mean production rate of 1.9 ± 3.34 μl in the first day, 1.58 ± 2.66 μl in the second day and 0.83 ± 2.05 μl in the third day, after which nectar production ceased completely. Based on the analysis of the daily nectar production, two groups of flowers were distinguishable in the sampled population when taking into account the beginning of nectar release. In 58% of nectariferous flowers, nectar release started just before flower opening (**Figure 4C**, from now on named ‘early’ flowers). In these flowers, the maximum volume of nectar occurred at 0 h, followed by the addition of smaller amounts of nectar until 24 h and by production cessation (*F*_6,59.81_ = 9.4, *P* < 0.001) (**Figure 4D**). In the other 42% of nectariferous flowers, nectar release started only by the end of the first day of anthesis (**Figure 4E**, from now on named ‘late’ flowers), with the maximum volume of nectar registered at 24 h, followed by a smaller production until 48 h and by production cessation (*F*_6,15.1_ = 25.66, *P* < 0.001) (**Figure 4F**). It is noteworthy that, regardless of the day that production started, each flower released nectar during the maximum of 2 days (**Figures 4C,E**). After the discovery that *J. oxyphylla* presented these two distinct nectar production rhythms, we verified if ‘early’ and ‘late’ flowers produced similar volumes of nectar during their first day of nectar production (0 h for ‘early’ flowers and 24 h for ‘late’ ones), and during their second day of production (24 h for ‘early’ flowers and 48 h for ‘late’ ones). For that, we performed Wilcoxon Rank Sum test with continuity correction for unbalanced samples. We observed that the volumes of nectar produced by both flower types were similar in the first and second days of nectar production (*W* = 86, *P* = 0.8612, and *W* = 107.5, *P* = 0.2562, for first and second days of production, respectively).

**FIGURE 5:**
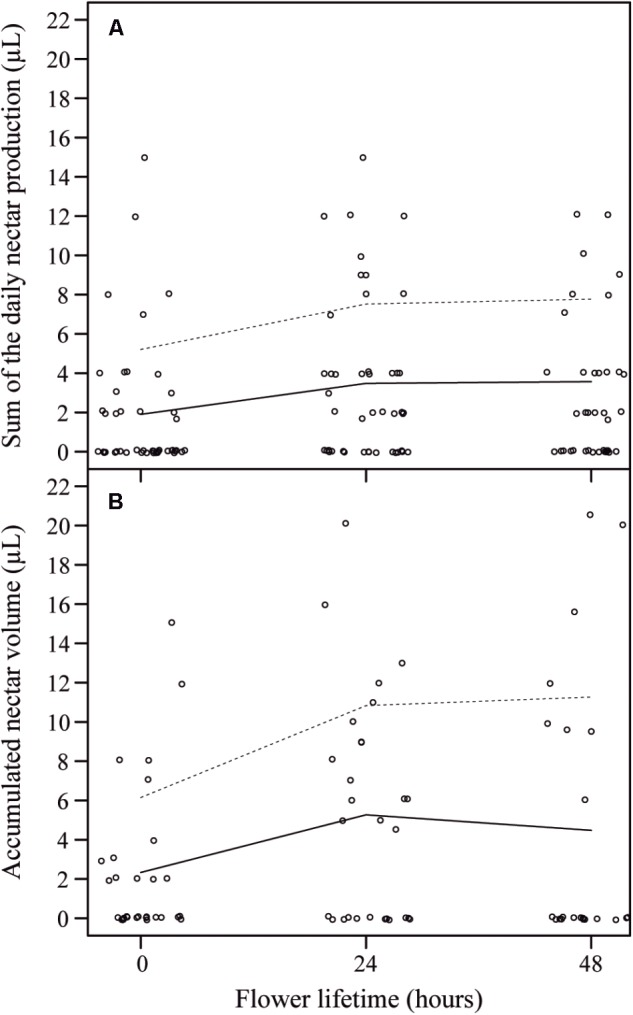
Sum of the daily nectar production per flower lifetime and accumulated nectar volume per flower lifetime in *J. oxyphylla* flowers. **(A)** Sum of the daily nectar production per flower lifetime. The trend lines describe a visual relationship between the two variables (nectar production rate and flower lifetime) based on the lowest smoother using a locally weighted regression (LOESS). Dashed lines are 95% confidence interval upper limits. The 95% confidence interval lower limits were zero and the line was omitted. **(B)** Accumulated nectar volume per flower lifetime. The trend lines also describe a visual relationship between the two variables (nectar production rate and flower lifetime) based on LOESS regression. There were no differences between the sum of the daily nectar production from 0 to 48 h and the accumulated nectar at 48 h of anthesis.

When we compared the initial overall volume of nectar produced (0 h) to the subsequent volumes produced by flowers (based on the sum of the daily nectar production rates), we observed that in the first day occurred the highest nectar production (from 0 h to 24 h), followed by an insignificant addition of nectar in the subsequent day (from 24 to 48 h), so that the sum of the volumes at 48 h was similar to the sum of the volumes at 24 h of anthesis [X^2^_(2)_ = 6.93, *P* = 0.031] (**Figure 5A**). Even though we found a high variation in nectar volume when comparing flowers and plants, the overall accumulated amount of nectar per day was similar throughout the first 48 h of anthesis [X^2^_(2)_ = 5.37, *P* = 0.068] (**Figure 5B**). Additionally, nectar mean concentration (25.91 ± 6.32% w/w) and the total milligrams of sugar per flower (1.35 ± 1.02 mg S) remained constant throughout this period (*F*_3,89.42_ = 1.91, *P* = 0.162; *F*_3,39_ = 1.34, *P* = 0.277, respectively). Finally, we did not observe any difference when comparing the accumulated nectar at 48 h of anthesis with the sum of the daily nectar production from 0 to 48 h (*W* = 456.5, *P* = 0.406).

### Comparative Histological and Cellular Analyses

#### Nectariferous and Nectarless Disks’ Histology and Histochemistry

**FIGURE 6:**
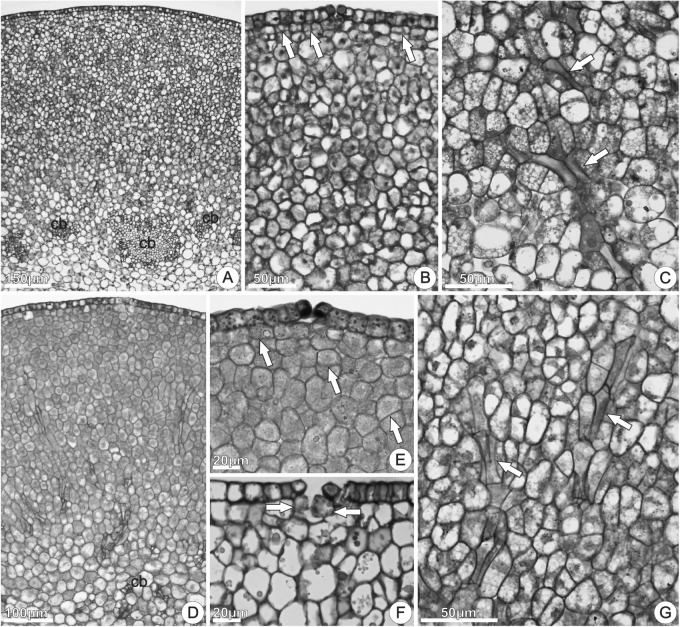
Structure of the floral disk of *Jacaranda oxyphylla*, illustrated by cross **(A,B,D,E,G)** and longitudinal **(C,F)** sections. **(A–C)** Nectariferous flowers; **(D–G)** nectarless flowers. **(A)** General aspect of the disk showing the epidermis, nectary parenchyma, sub-nectary parenchyma and collateral bundles; **(B)** detail of **(A)** showing epidermis coated with thin cuticle, stomata, and nectary parenchyma region composed by isodiametric cells. Arrows indicate cell division in the subepidermal layers; **(C)** subnectary parenchyma with phloem strands (arrows); **(D)** general aspect of the disk showing the epidermis, parenchyma, and vascular tissues; **(E)** epidermal cells with dense globules and stomata with associated secretion. Arrows indicate cell division in the subepidermal layers; **(F)** detached epidermal cells and large parenchyma cells inside substomatic chamber (arrows); **(G)** phloem strands (arrows) in the parenchyma tissue. cb, collateral bundles.

The disk volume in nectariferous flowers (5.50 ± 2.25 mm^3^) and in nectarless flowers (5.60 ± 2.11 mm^3^) was similar (*t*_14.24_ = -0.0998, *P* = 0.922). Disks from both nectariferous (**Figures 6A–C**) and nectarless flowers (**Figures 6D–G**) at 0 h of anthesis, in cross sections through the median region, were constituted by uniseriate epidermis with stomata (**Figure 6B,E**) across the entire disk surface and several layers of parenchyma vascularized with only phloem (**Figures 6C,F**). Although both disks exhibited a similar histological organization, nectariferous flowers showed more clearly two parenchyma regions, nectary and subnectary parenchyma.

In nectariferous flowers, the nectary parenchyma (underlying the epidermis) was composed by several layers of small, isodiametric, thin-walled cells, with relatively large nucleus, dense cytoplasm, and developed vacuoles (**Figures 6A,B**). The subnectary parenchyma, in continuity with the nectary parenchyma, was composed by larger cells, with irregular shapes, less dense cytoplasm, and larger intercellular spaces (**Figure 6A**). Phloem strands coming from the collateral bundles ramify into the subnectary parenchyma (**Figure 6C**). Parenchyma cells in division were commonly observed in both nectariferous and nectarless flowers (**Figures 6B,E**).

In nectarless flowers (**Figures 6D–G**), the epidermal cells had irregular sizes and shapes and numerous globules in the protoplast (**Figure 6E**). Larger, vacuolated and irregularly shaped parenchyma cells, located just under the stomata, expanded toward the substomatic chamber and kept interspersed among the epidermal cells (**Figure 6F**). Stomata with enlarged aperture, and loose or detached epidermal cells were commonly observed (**Figure 6F**). Comparing with nectariferous flowers, the parenchyma region presented lower number of layers composed by juxtaposed cells (**Figures 6D,E**), with small intercellular spaces, vascularized with phloem strands (**Figure 6G**).

**FIGURE 7:**
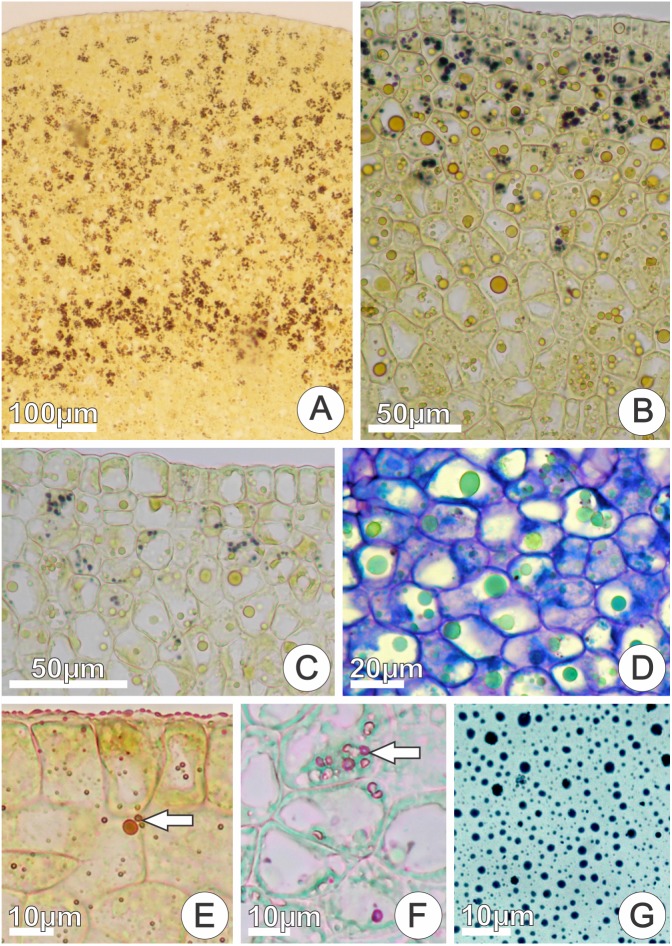
*In situ* location of the main classes of the chemical compounds detected in cross sections of nectariferous disks and in nectar of *Jacaranda oxyphylla* flowers. **(A–C)** Positive reaction to Lugol’s iodine showing progressive depletion of starch grains during anthesis (0, 24, 48 h flowers, respectively); **(D)** phenolic inclusions (in green) with toluidine blue staining (48 h flowers); **(E)** positive reaction to Sudan IV for lipids (arrow) (48 h flowers); **(F)** positive reaction to NADI’s reagent for essences (arrow) (48 h flowers); **(G)** positive reaction for lipids in raw floral nectar with Sudan Black B (48 h flowers).

Starch grains, phenolic substances, lipid bodies, and essences were detected in both nectariferous and nectarless 48 h flowers. A clear decrease in the amount of starch grains (**Figures 7A–C**) occurred at the begging of anthesis of nectariferous and nectarless flowers (0–48 h). On the other hand, phenolic substances (**Figure 7D**), lipid bodies (**Figure 7E**), and essences (**Figure 7F**) became more abundant throughout anthesis in both flower types. Sudan Black B reacted positively for lipids in nectar (**Figure 7G**). The results of the histochemical tests on the secretory disk of nectariferous and nectarless flowers of *Jacaranda oxyphylla* are summarized in the **Table 1**.

**Table 1:** Histochemical tests on the secretory disk in nectariferous and nectarless flowers of *Jacaranda oxyphylla* (Bignoniaceae).

Staining procedure	Target compounds	Positive reaction site	
		
		Nectariferous flowers	Nectarless flowers
Sudan IV	Total lipids	Cuticle, subcuticular space, cell wall, cytoplasm, and vacuole (EP, NP, SN)	Cuticle, subcuticular space, cell wall, cytoplasm, and vacuole (EP, NP, SN)
Ferric chloride	Phenolic compounds	Amyloplasts and vacuole (EP, NP, SN)	Amyloplasts and vacuole (EP, NP, SN)
Lugol’s iodine	Starch	Amyloplasts and vacuole (EP, NP, SN)	Amyloplasts and vacuole (EP, NP, SN)
NADI’s reagent	Essences	On the cuticle, subcuticular space, cytoplasm, vacuole (EP, NP, SN)	On the cuticle, subcuticular space, cytoplasm, vacuole (EP, NP, SN)


*EP, epidermis; NP, nectary parenchyma; SN, sub-nectary parenchyma.*

#### Nectariferous and Nectarless Disks’ Ultrastructure

We investigated the subcellular organization of nectariferous and nectarless flowers in *J. oxyphylla* with emphasis on plastid changes, considering the flower life stages in which we observed the presence of nectar (0–48 h of anthesis) and after nectar production cessation (72 h of anthesis).

##### Nectariferous flowers

###### ‘Early’ flowers: flowers that started releasing nectar at 0 h of anthesis

**FIGURE 8:**
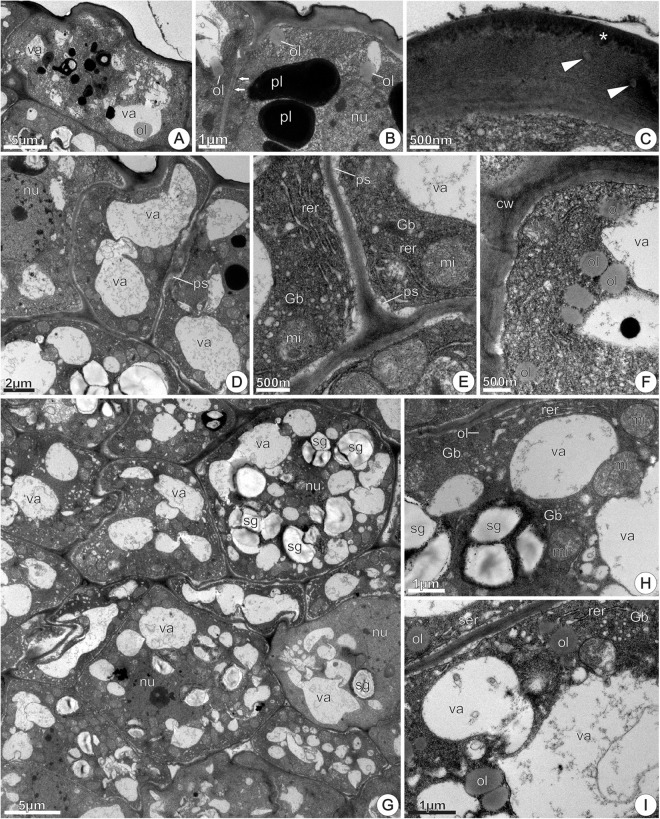
TEM micrographs of the disk from *Jacaranda oxyphylla* nectariferous flowers, at 0 h of anthesis. **(A–F)** Epidermal cells. **(A)** Rectangular cells with cytoplasm and vacuoles with heterogeneous inclusion; **(B)** plasmodesmata (arrows), conspicuous nucleus, abundant cytoplasm with dense plastids and oil drops; **(C)** detail of the outer tangential cell wall highlighting oil inclusions (arrow heads) embedded in the wall matrix and osmiophilic deposits (asterisk) in the cuticular layer; **(D)** conspicuous nucleus, developed vacuoles, sinuous plasmalemma, and small periplasmic spaces; **(E)** detail of **(D)** showing abundance of organelles in the peripheral cytoplasm and flocculent materials inside vacuole and periplasmic space**; (F)** polyribosomes through the cytosol and oil bodies adjacent to tonoplast. **(G–I)** Nectary parenchyma. **(G)** General aspect showing cells side by side with different ultrastructure; **(H)** detail of **(G)** showing amyloplasts with residual starch grains and RER profiles assembled around degenerating amyloplasts and vacuoles; **(I)** vacuoles with flocculent materials and membrane debris, hyperactive Golgi body, enlarged SER elements and oil drops. Gb, Golgi body; mi, mitochondria; nu, nucleus; ol, oil; pl, plastid; ps, periplasmic space; rer, rough endoplasmic reticulum; ser, smooth endoplasmic reticulum; sg, starch grains; va, vacuole.

Nectary disk from recently opened flowers, at 0 h of anthesis, showed rectangular epidermal cells with voluminous nuclei, dense cytoplasm and little-developed vacuoles containing osmiophilic bodies, flocculent material, oil drops, and membrane debris (**Figure 8A**). Plasmodesmata connected epidermal cells with each other and with the underlying parenchyma (**Figure 8B**). The outer tangential walls were thick, sinuous and covered with a thin, smooth cuticle (**Figure 8B**), which was composed of an inner reticulate layer containing microchannels and an outer amorphous layer that corresponded to the cuticle proper; osmiophilic deposits occurred in the cuticle layer and oil inclusions in the cell wall matrix (**Figure 8C**). The cuticle was continuous and did not have cracks, tears, or pores (**Figures 8B,C**). Large nuclei, free ribosomes, rough endoplasmic reticulum (RER), mitochondria and plastids characterized the epidermal cells in this stage (**Figure 8B**). Plastids, with residual starch grains (**Figure 8A**) or lacking starch (**Figure 8B**) had very electron-dense, homogenous stroma due to phenolic substances accumulations. Oil drops occurred inside vacuoles (**Figure 8A**), close to the plasma membrane or juxtaposed to the tonoplast (**Figure 8B**). At the same stage, sections of the central region of the disk showed epidermal cells with greater development of vacuoles, sinuous plasmalemma and periplasmic spaces (**Figure 8D**), besides cytoplasm with more evident organelles, especially RER, mitochondria and Golgi bodies (**Figure 8E**). The RER profiles were extensive, exhibited dilated regions and were generally situated in the peripheral cytoplasm, adjacent to the plasma membrane (**Figure 8E**). There were many vesicles near the swollen edges of the RER and their location and arrangement suggested that they had budded off from the RER profiles. Moreover, images suggested the fusion of coated vesicles with the plasma membrane, which showed sinuous contour (**Figure 8E**). Accumulations of flocculent material occurred inside vacuoles (**Figure 8D**) and in periplasmic spaces (**Figure 8E**). Oil bodies occurred scattered in the cytosol, near the plasmalemma or tonoplast (**Figure 8F**). In the same section of the disk, nectary parenchyma cells located side by side exhibited different ultrastructure (**Figure 8G**). In some cells, amyloplasts exhibited reduced starch grains with hydrolysis signals, or residues of starch grains (**Figure 8G**, top right corner). Some cells had small nucleus, numerous undifferentiated vacuoles and dense cytoplasm (**Figure 8G**) with mitochondria and RER profiles, which were more commonly located near the degenerating plastids or surrounding the vacuoles containing flocculent materials (**Figure 8H**). Other neighboring cells showed conspicuous nucleus with evident nucleolus, denser and abundant cytoplasm and merged vacuoles (**Figure 8G**). Amyloplasts were absent in these cells. Flocculent material, probably originated from starch hydrolysis, was incorporated into the cytoplasmic matrix (**Figures 8G,I**). The vacuoles had irregular sizes and shapes and progressively merged with each other forming larger vacuoles (**Figures 8G,I**). Increase in cytoplasmic density and in the amount of mitochondria and oil bodies, besides the appearance of smooth endoplasmic reticulum (SER) with dilated elements characterized these cells (**Figures 8G,I**). SER elements occurred mainly located in the peripheral cytoplasm (**Figure 8I**).

**FIGURE 9:**
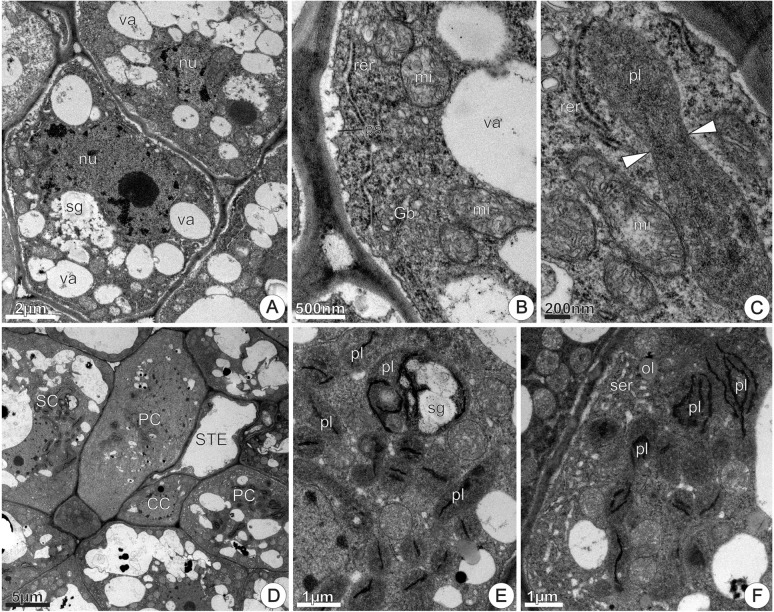
TEM micrographs of the disk from *Jacaranda oxyphylla* nectariferous flowers, at 24 h of anthesis. **(A–C)** Nectary parenchyma. **(A)** General aspect showing vacuolated cells with prominent nucleus and evident nucleolus, degenerating amyloplast and merged vacuoles; **(B)** detail of **(A)** showing cytoplasm rich in polyribosomes, mitochondria, extensive RER and Golgi bodies located in the peripheral cytoplasm, and developed periplasmic spaces; **(C)** elongated plastid with clear median constriction (arrowheads) surrounding with RER elements and mitochondria. **(D–F)** Subnectary parenchyma. **(D)** General view of the subnectary parenchyma and phloem cells; **(E)** detail of (D) showing chloro-amyloplasts and undifferentiated plastids in parenchyma cell; **(F)** plastids with thylakoid-like membranes and dilated SER elements assembled in the peripheral cytoplasm of phloem parenchyma cell. CC, companion cell; Gb, Golgi body; mi, mitochondria; nu, nucleus; ol, oil; PC, phloem parenchyma cell; pl, plastid; ps, periplasmic space; rer, rough endoplasmic reticulum; ser, smooth endoplasmic reticulum; sg, starch grains; SC, subnectary parenchyma cell; STE, sieve tube element; va, vacuole.

At 24 h of anthesis, most of the nectary parenchyma cells exhibited a similar pattern to the observed at the previous stage, characterized by vacuoles containing flocculent materials and few residual starch grains (**Figure 9A**). Moreover, these cells exhibited conspicuous nucleus with evident nucleolus and dense cytoplasm (**Figure 9A**) rich in polyribosomes, mitochondria, Golgi bodies and extensive RER together vesicles located in the peripheral cytoplasm (**Figure 9B**). The plasmalemma was sinuous in outline and periplasmic spaces contained flocculent materials (**Figure 9B**). At this stage, the occurrence of a distinct plastid type, not observed at previous stages, was remarkable. It featured an elongated shape, granular stroma with small lipid droplets and an irregular, poorly developed inner membrane system (**Figure 9C**). The presence of a narrow constriction in their middle region is noticeable and is an evidence of plastid division. Mitochondria and RER profiles were common around these plastids (**Figure 9C**).

In the sub-nectary region, the parenchyma cells associated or not with phloem, were characterized by a developed vacuole system (**Figure 9D**). Parenchyma cells in this nectary region exhibited numerous undifferentiated chloroplasts through the cytosol, and some of them contained small starch grains and few developed thylakoids (**Figure 9E**). Phloem parenchyma cells in this region also had undifferentiated chloroplasts with thylakoid-like membranes, small vacuoles and abundant SER elements situated in the periphery of the cytoplasm (**Figure 9F**).

**FIGURE 10:**
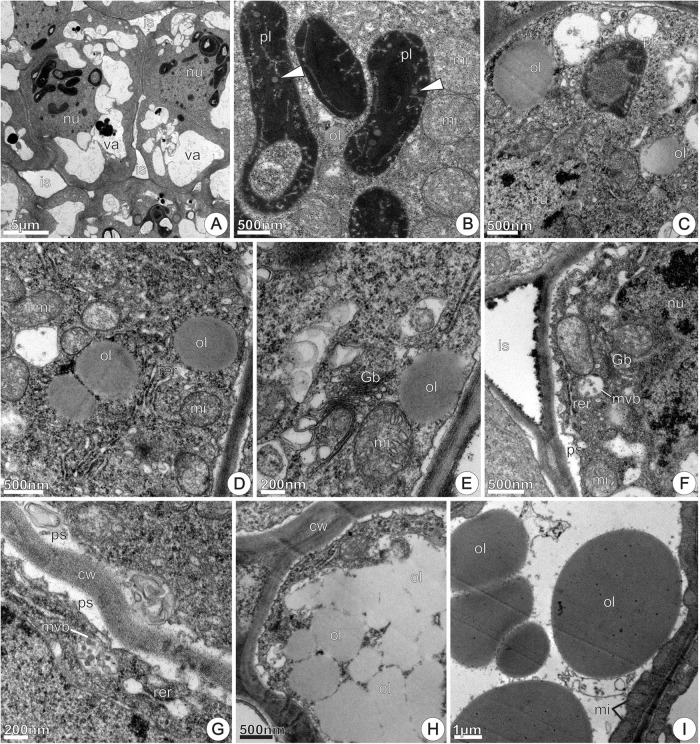
TEM micrographs of the disk from *Jacaranda oxyphylla* nectariferous flowers, at 48 h of anthesis. **(A)** General view of the nectary parenchyma showing larger intercellular spaces and parenchyma cells with prominent nucleus and dense cytoplasm; **(B)** polymorphic plastids with electron-dense stroma, vesicular/tubular inner membranes and lipid globules; **(C)** large oil droplets, RER elements and mitochondria scattered through the cytosol; **(D)** part of a nectary parenchyma cell highlighting the conspicuous nucleus, modified plastid and oil droplets; **(E)** oil drop near the plasmalemma, numerous polyribosomes and hyperactive Golgi body; **(F)** mitochondria and RER elements positioned in the peripheral cytoplasm, sinuous plasmalemma and dense granulations inside intercellular space; **(G)** part of two nectary parenchyma cells highlighting multivesicular body in close juxtaposition with the plasmalemma that is sinuous, and developed periplasmic space containing dense granulations and multilamellar membranes; **(H)** assemblage of oil drops in the cytoplasm; **(I)** large oil bodies in the vacuole, and clustered mitochondria in the reduced parietal cytoplasm. cw, cell wall; Gb, Golgi body; is, intercellular space; mi, mitochondria; mvb, multivesicular body; nu, nucleus; ol, oil; pl, plastid; ps, periplasmic space; rer, rough endoplasmic reticulum; sg, starch grains; va, vacuole.

At 48 h of anthesis, nectary parenchyma region had more developed intercellular spaces (**Figure 10A**) when compared to the previous stage. In this stage, there was a remarkable occurrence of polymorphic plastids (**Figures 10A,B**) featured by electron-dense stroma, small oil globules and vesicle/tubular inner membranes. The richness in free ribosomes, large mitochondria, SER and RER profiles was noticeable (**Figures 10B–F**), in addition to the considerable increase in number and size of lipid bodies in the cytoplasm (**Figures 10C–E**). In the region of intercellular spaces, cells had organelles located in parietal position; dense granulations occurred adhered to the cell walls, bordering the intercellular space (**Figure 10F**). Across the entire cell surface occurred multivesicular bodies and dilated profiles of RER near the plasmalemma, besides periplasmic space, which was prominent and contained multilamellar membranes and dense granulations (**Figure 10G**). We observed oil drops close to the plastids (**Figure 10B**), scattered (**Figures 10C,D**) or clustered (**Figure 10H**) in the cytoplasm, close to the plasmalemma (**Figure 10E**), and inside vacuoles (**Figure 10I**), where they merged together forming conspicuous oil bodies. At 72 h of anthesis, parenchyma cells had similar features (not shown here).

###### ‘Late’ flowers: flowers that started releasing nectar at 24 h of anthesis

At 0 h of anthesis, disks from ‘late’ flowers presented similar features to those from ‘early’ flowers at 0 h of anthesis, showing abundance of amyloplasts and beginning of starch grains hydrolysis.

At 24 h of anthesis, nectaries disks from ‘late’ flowers presented similar features as to those from ‘early’ flowers at 24 h of anthesis showing total or partial depletion of starch grains.

At 48 h, the nectaries from ‘late’ flowers presented similar features as those from ‘early’ flowers at 48 h of anthesis, with total depletion of starch grains and incorporation of the amyloplasts residues into the cytoplasmic matrix, changes in plastid type and an increase of lipid inclusions.

##### Nectarless flowers

**FIGURE 11:**
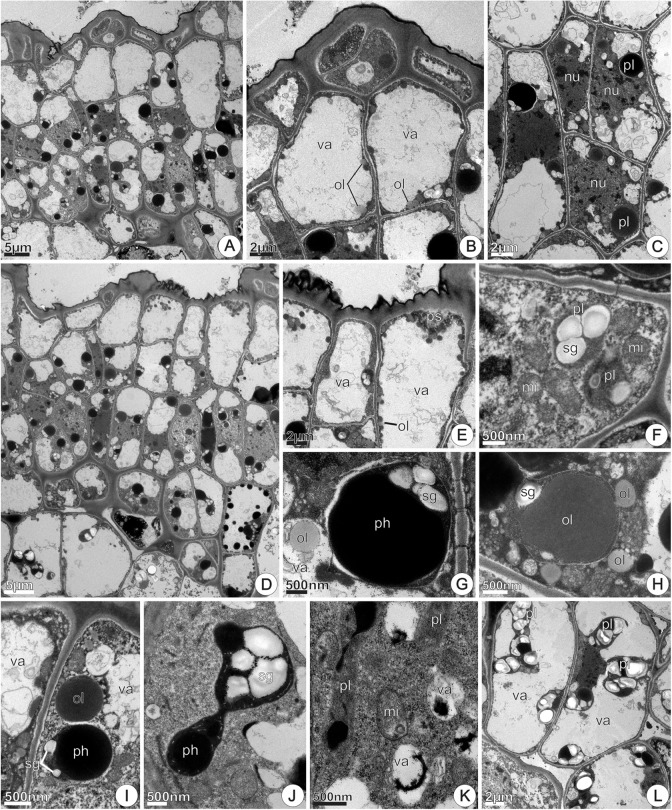
TEM micrographs of the disk from *Jacaranda oxyphylla* nectarless flowers. **(A–C,F)** At 0 h of anthesis. **(A)** General view showing irregular epidermis and juxtaposed subepidermal cells; **(B)** detail of **(A)** highlighting epidermal cells with dense cytoplasm, highly vacuolated subepidermal cells and osmiophilic inclusions facing the inner surface of the tonoplast; **(C)** cluster of three newly derivate subepidermal cells showing voluminous nucleus and rounded plastids with dense inclusions. **(D,E,G–L)** At 24 h of anthesis. **(D)** General view showing vacuolated parenchyma cells interspersed with detached epidermal cells; **(E)** detail of **(D)** showing osmiophilic droplets inside vacuole and periplasmic space besides secretions on the cuticle; **(F)** amyloplast with globular starch grains and large mitochondria; **(G)** rounded plastid containing small starch grains and phenolic content, and lipid drop in the vacuole; **(H)** oval plastid filled with lipid content from which oil drops flow toward the cytoplasm; **(I)** large oil drop near plastid with reduced starch grains; **(J)** dimorphic plastid with starch grains and phenolic content in the opposite poles, showing a constriction in their median region; **(K)** undifferentiated plastids and vacuoles containing dense inclusions; **(L)** general view of subnectary parenchyma showing vacuolated cells with prominent amyloplasts. mi, mitochondria; nu, nucleus; ol, oil; pl, plastid; ph, phenolic content; sg, starch grains; va, vacuole.

At 0 h of anthesis, the epidermal and parenchyma cells exhibited variable sizes and shapes, and variable cytoplasmic densities (**Figure 11A**). Epidermal cells were thick-walled and covered with a continuous thin cuticle. In the cytoplasm, SER profiles and mitochondria were the most evident organelles (**Figure 11B**). The first subepidermal parenchyma layer consisted of thin-walled expanded cells, with cytoplasm reduced to a thin parietal layer (**Figure 11B**). Amyloplasts were uncommon in these cells, while lipophilic inclusions were abundant and occurred adhered to the inner surface of the tonoplast in epidermal and subepidermal cells (**Figure 11B**). The subsequent two to three parenchyma layers were composed by axially elongated cells that differed from those of the first subepidermal layer regarding cytoplasmic density and vacuole system development. In this disk region, clusters of two to three cells with smaller sizes and characterized by thinner walls, prominent nucleus, abundant cytoplasm, and poorly developed vacuoles were common (**Figure 11C**). The occurrence of small amyloplasts with prominent starch grains and large mitochondria was common in newly derived cells (**Figure 11F**).

At 24 h of anthesis, the most remarkable difference in relation to the previous stage was the occurrence of protuberances on the epidermal cells’ outer tangential walls (**Figures 11D,E**). Moreover, large cells that at the previous stage were located in the subepidermal position (**Figures 11A,B**), now appeared interspersed with epidermal cells. This aspect was also observed in histological sections (**Figure 6F**). In addition, osmiophilic materials were more abundant at this stage and could be observed on the cuticle surface and mainly in periplasmic space (**Figure 11E**).

Subepidermal parenchyma cells, at both 0 and 24 h of anthesis, had similar ultrastructural organization, characterized by scarce amyloplasts with few or lacking starch grains. The coexistence of plastids with distinct morphologies and/or inclusions was common in the same or neighboring cells. The most common type of plastids was rounded, devoid of thylakoids, filled with electron-dense phenolic content and containing small starch grains (**Figures 11C,G,I**). Oval-shaped plastids with reduced or lacking starch grains and large oil inclusions from which oil drops flow toward the cytoplasm and vacuoles were also observed (**Figure 11H**). Oil drops occurred near the plastids (**Figure 11I**). Dimorphic plastids having conspicuous pressed starch grains on one of its poles and, vesicle/tubular membranes on the opposite pole devoid of starch grains (**Figure 11J**), occurred in the interface between subepidermal and deeper parenchyma layers. Elongated, undifferentiated plastids were common in these cells (**Figure 11K**). In the deeper parenchyma layers (**Figure 11L**), all the plastids contained conspicuous starch grains and were larger than those in subparenchyma layers.

**FIGURE 12:**
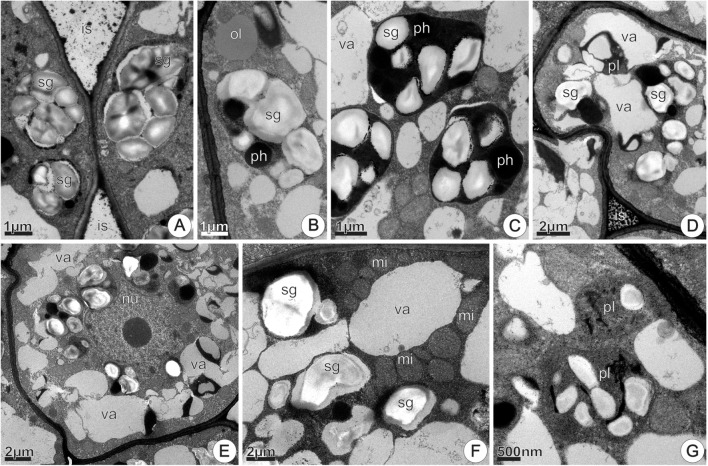
TEM micrographs of the disk from *Jacaranda oxyphylla* nectarless flowers, at 48 h of anthesis, highlighting the plastids change in the deeper parenchyma layers. **(A)** Bigger amyloplasts in the periphery of the cell and black granulations adhered in the cell walls bounding the intercellular space; **(B)** amyloplast with voluminous starch grains and phenolic inclusions. Note oil drop in the cytosol; **(C)** denser amyloplasts with signs of starch hydrolysis; **(D)** degenerating amyloplast engulfed in the vacuole; **(E)** merged vacuoles from amyloplasts degeneration in the periphery of the cell, and amyloplasts clustered around the nucleus; **(F)** mitochondria assembled around vacuoles; **(G)** Chloro-amyloplasts with undeveloped thylakoids. is, intercellular space; mi, mitochondria; nu, nucleus; ol, oil; pl, plastid; ph, phenolic content; sg, starch grains; va, vacuole.

At 48 h of anthesis, the features of the subepidermal layers remained similar to the observed at the previous stage (not shown here). The most remarkable difference occurred in the deeper parenchyma layers, which showed an increase in the amount and volume of starch grains in plastid profiles (**Figures 12A–C**), besides accumulations of black granulations in the intercellular space (**Figure 12A**). In addition, parenchyma cells exhibited dense cytoplasm and large oil bodies (**Figure 12B**). The progressive increase in accumulations of phenolic compounds in the amyloplasts was also noticeable (**Figures 12B–D**). Signs of amyloplasts degeneration and hydrolysis of the starch grains were commonly observed, and plastids debris, including phenolic compounds, were seem inside vacuoles (**Figure 12D**). The vacuoles merged together forming larger vacuoles on cell periphery (**Figure 12E**), pushing the remaining amyloplasts toward the nucleus, which occupies a central position. Also, in these cells, clusters of globular mitochondria around the vacuoles or degenerating amyloplasts were common (**Figure 12F**). Chloro-amyloplasts with undeveloped thylakoids were commonly found at this stage (**Figure 12G**). These plastids contained small globular starch grains, abundant stroma and were devoid of phenolic compounds.

### Volatile Compounds Common to Floral Disk and Floral Scent

The chemical analyses of the floral disks revealed that the disks of nectariferous flowers had no volatile compounds in common with *in situ* floral scent. In contrast, the disks of nectarless flowers presented four volatile compounds that were also present in *in situ* floral scent. Three of them were the aliphatic compounds tridecane, tetradecane, and hexadecane, and one was the aromatic compound phenylacetaldehyde (**Table 2**). We registered a variation in the presence of these compounds in the headspace samples, with one sample showing all the four compounds, two samples showing only phenylacetaldehyde and tridecane, one sample showing only tetradecane and hexadecane and one showing none of them.

**Table 2:** Absolute amounts (mean ± SE) of scent compounds found in *Jacaranda oxyphylla* (Bignoniaceae) floral disks (ng.disk^-1^) and *in situ* floral headspace (ng.flower^-1^; 30 min^-1^).

		Floral disks		
			
Compounds	RI	Nectarless	Nectariferous	Flowers
***Aliphatic compounds***				
Tridecane	1300	23.54	–	0.36 ± 0.04
Tetradecane	1400	29.25	–	0.63 ± 0.05
Hexadecane	1600	64.14	–	0.69 ± 0.49
***Aromatic compounds***				
Phenylacetaldehyde	1045	63.73	–	0.65 ± 0.23


*RI, Kovats retention index.*

## Discussion

In this study, we characterized the spatial and temporal variation in nectar production and compared disk histology, chemistry and cellular features in nectariferous and nectarless flowers, which corresponded to 53 and 47% of *J. oxyphylla* flowers, respectively. We found a broad spatial variation in nectar volume in nectariferous flowers, including intra- and inter-plant differences. Additionally, we also found temporal variation in nectar production, with 31% of the nectariferous flowers presenting the higher nectar volume at the moment of flower opening (‘early’ flowers) and 22% presenting it only in the second day of anthesis (‘late’ flowers). Both nectariferous flower types, ‘early’ and ‘late,’ exhibited cellular apparatus typical of nectar secretion, showing a continuous decrease of starch grains’ size and number during the first 48 h of anthesis. Although nectariferous and nectarless flowers showed similar histological organization, at cellular level, nectarless flowers exhibited osmophoric features. In fact, the disks of nectarless flowers showed volatile compounds that were also present in floral scent of *J. oxyphylla*, suggesting its participation in floral chemical signaling. In addition, disks in nectariferous flowers seem to play a dual function, secreting predominantly nectar in the first 48 h of anthesis and only lipophilic substances from this time on.

### Nectar Production Variability in Space and Time

Nectar volume variation at plant or population level, in general, could be caused by the nectarivores, which would empty flowers as they forage, or by plant species characteristics (Heinrich, 1975; Feinsinger, 1978; Brink and deWet, 1980; Zimmerman, 1981), such as the variable presence of nectariferous and nectarless flowers among plants (Gervasi and Schiestl, 2017). Both cases create heterogeneity in the resource availability to pollinators in natural populations, in a way that pollinators could or could not react to it (Real, 1981). The nectar variability reported in this study could be responsible for the scarce pollinator visits and, consequently, for the low reproductive success described for *J. oxyphylla* by Guimarães et al. (2008). On the other hand, nectarless flowers could favor cross-pollination, as they may coerce pollinators to visit other plants after encountering some empty flowers (Thakar et al., 2003). This scenario could be especially relevant in a self-incompatible species, such as *J. oxyphylla* (Guimarães et al., 2008) since it may reduce geitonogamy and favor allogamy (Johnson, 2000).

The occurrence of nectarless flowers has been associated with high-density plant populations (Thakar et al., 2003; Anand et al., 2007; Zhao et al., 2016), which is the case of *J. oxyphylla* that showed clumped distribution in the study natural population. The presence of nectarless flowers in natural populations may represent advantages from the plants’ perspective (Thakar et al., 2003 and references therein), especially, in plant species with concealed nectar, as *J. oxyphylla*, which are most likely to present highly variable nectar volume (Bell, 1986). As pollinators have no visual cue to predict the presence or absence of nectar in a flower before trying it (Smithson and Gigord, 2001), it is expected that they would not exert selective pressures toward nectar volume stabilization. Therefore, these species might present a higher proportion of flowers without nectar or with very low volumes, supporting the idea that those plant species cheat on pollinators (Bell, 1986; Gilbert et al., 1991). In fact, even though some Bignoniaceae species have complex floral nectaries (Lopes et al., 2002; Machado et al., 2017b) and produce large amounts of nectar (Cruden et al., 1983), others have no nectaries (Alcantara and Lohmann, 2010) or nectar, being pollinated by deceit (Umaña et al., 2011). However, such an expressive intraspecific variation in nectar production has never before been reported for any Bignoniaceae species.

A variety of plant species, which are known to produce nectarless flowers, are believed to be pollinated through Batesian mimicry (Firmage and Cole, 1988; Johnson, 1994, 2000; Gigord et al., 2002; Juillet et al., 2007). Additionally, nectarless species could benefit from cheating on naïve pollinators (Gigord et al., 2002). The mimicry idea could also apply to plant species that present individuals having only nectariferous flowers and only nectarless flowers in the same population, or even mixed proportions of nectariferous and nectarless flowers in the same individuals, as does *J. oxyphylla*. Plants that present only nectarless flowers could have lower reproductive success when compared to plants with only nectariferous flowers and with mixed flower types, because pollinators can learn to avoid them (Smithson and MacNair, 1997; Ferdy et al., 1998; Gumbert and Kunze, 2001; Vásquez and Barradas, 2018). Besides the heterogeneity of nectar production within and among plants in *J. oxyphylla*, our results also showed high heterogeneity among nectariferous flowers as well, which is showcased by the fact that we found two types of nectar production rhythms (‘early’ and ‘late’ flowers). The causes of this variation in nectar production rhythm in *J. oxyphylla* flowers are yet unknown. Most bee-pollinated Bignoniaceae species start nectar production before anthesis (Galetto, 1995; Lopes et al., 2002; Maués et al., 2008; Guimarães et al., 2016; Quinalha et al., 2017; Souza et al., 2017), so that pollinators have high probability of finding nectar in freshly opened flowers. However, in *J. oxyphylla*, when searching for nectar in recently opened flowers, pollinators have a 78% chance of finding empty flowers, considering that 47% of flowers are nectarless and 31% start nectar release just in the second day of anthesis (‘late’ flowers).

Nectar reabsorption is a common phenomenon in angiosperms (Torres and Galetto, 1998; Stpiczyńska, 2003; Nepi and Stpiczyńska, 2008; Antoń et al., 2017), which has been considered as resource-recovery strategy (Nepi and Stpiczyńska, 2008). One might have thought that the occurrence of nectarless flowers in *J. oxyphylla* would be a sign of nectar reabsorption; however, our results showed that there was no decrease in the accumulated nectar volume throughout anthesis and no difference between the accumulated volume and the sum of daily nectar production. These findings together with the wide window of monitoring flowers (144 h) showed that, undoubtedly, nectarless flowers have no nectar from start to finish of anthesis, and that nectar reabsorption is not the cause of it.

### Histological, Histochemical, and Ultrastructural Features of the Floral Disk

According to our results, there are no significant histological and histochemical differences between the disks of nectariferous and nectarless flowers of *J. oxyphylla*. However, ultrastructural observations allowed us to identify differences concerning their fine structure and functioning. Our histochemical and utrastructural analysis suggest that the disk of nectariferous flowers has a dual function, wherein until 48 h of anthesis it produces predominantly nectar and in subsequent stages of anthesis, lipophilic secretion is predominant. Moreover, the ultrastructural analysis provided clear evidence to conclude that the disk of nectarless flowers has secretory activity associated with lipophilic secretion during the whole anthesis.

The disk in nectariferous flowers of *J. oxyphylla* showed typical characteristics of nectary tissues, such as small thin-walled cells, large nuclei, small vacuoles and dense cytoplasm (Fahn, 1979; Nepi, 2007; Guimarães et al., 2016). The ultrastructural features observed in nectariferous flowers at the beginning of anthesis (at 0 and 24 h) are similar to those reported for nectaries of other angiosperms and are indicative of high metabolic activity (Fahn, 1979; Nepi, 2007; Guimarães et al., 2016; Machado et al., 2017b). The subcellular changes observed in these flowers at this moment of anthesis, mainly the alterations in amyloplasts, are involved in the conversion of starch to nectar, as reported for other angiosperm species (e.g., Fahn and Shimony, 2001; Nepi, 2007; Paiva and Machado, 2007; Guimarães et al., 2016; Machado et al., 2017b). The juxtaposition of large mitochondria with amyloplasts may be related to energy requirements during starch hydrolysis (Fahn, 1979). The occurrence of polyribosomes, well-developed Golgi bodies and RER elements can also be related to the synthesis of enzymes involved in starch grains’ hydrolysis and degradation processes, which were observed during nectar secretion. Moreover, RER elements may also be involved in translocation and/or temporary concentration of sugars (Durkee, 1983; Figueiredo and Pais, 1992; Paiva and Machado, 2007). The occurrence of vesicles close or fused to plasmalemma, and the formation of ample periplasmic spaces suggest that the elimination of secretion products from the protoplast occurs by exocytosis (Fahn, 1979; Nepi, 2007; Machado et al., 2017b).

Our data indicate that the production of lipophilic substances increased throughout the anthesis in nectariferous flowers. This data is compatible with the predominance of elaioplasts in the nectary parenchyma cells at 48 h of anthesis, when most of amyloplasts are degenerated and nectar secretion has already stopped. Plastid change occurred simultaneously with the increase in the amount of oil drops inside vacuoles or dispersed in the cytosol, and SER proliferation, which are ultrastructural evidences of lipophilic secretion (Gleizes et al., 1980; Figueiredo and Pais, 1992; Turner et al., 1999; Machado et al., 2005; Stpiczyńska et al., 2005; Stpiczyńska and Davies, 2016; Possobom and Machado, 2018). Although these features are unusual in nectary tissues, the ability of nectary cells to produce both nectar and lipids (Baker and Baker, 1975; Durkee et al., 1984; Subramanian et al., 1990; Possobom et al., 2010; Tölke et al., 2015; Guimarães et al., 2016; Stpiczyńska and Davies, 2016; Machado et al., 2017b) and transition from a true nectary to a lipophilic secretory gland (Durkee, 1982; Durkee et al., 1984) has been reported in some angiosperm species.

Our histological and ultrastructural observations revealed that the disk from nectarless flowers had subcellular evidences of secretory activity associated to the production of volatile substances. The histological characteristics exhibited by the disk, as an irregular surface that enhances the area of secretion release, highly vacuolated epidermal cells, compact arrangement of subepidermal tissue with several layers of depth and vascularization with vein endings consisting of phloem only, are common to osmophores, according to Vogel (1990).

Some authors, studying Orchidaceae species (de Melo et al., 2010; Kowalkowska et al., 2012, 2015; Wiśniewska et al., 2018), found that floral nectaries and osmophores are somewhat similar in structure and ultrastructure features, except that in the latter there is predominance of SER and low frequency of Golgi bodies. Therefore, abundance of globular mitochondria, SER with peripheral distribution and oil droplets in the cytosol and in amyloplasts, together with the scarce Golgi bodies here observed may be associated with fragrance production (Curry et al., 1991; Stpiczyńska and Davies, 2016). The occurrence of polymorphic plastids containing many lipophilic droplets and numerous large oil inclusions in the cytoplasm of parenchyma cells both in nectariferous flowers at 48 h of anthesis and in nectarless flowers is a strong evidence of the involvement of the floral disk in scent production, as similar plastids also occur in osmophores (Pridgeon and Stern, 1983, 1985; Curry et al., 1991; de Melo et al., 2010; Antoń et al., 2012).

Although amyloplasts are common components of both nectaries and osmophores (Nepi, 2007), the absence of starch has been recorded in osmophores in some orchid species (Wiśniewska et al., 2018). Plastids lacking or having reduced starch grains in the epidermis and in the first subepidermal layers at recently opened nectarless flowers, as here observed, might be caused by their hydrolysis before anthesis (at an earlier stage than we sampled), as starch grains are utilized as energy source for scent production (Stern et al., 1987; Vogel, 1990; Nepi, 2007; Pacini and Nepi, 2007; de Melo et al., 2010).

Small droplets of lipophilic material in the disk epidermal cells that stained with Sudan IV and Nadi reagent, and also observed in TEM analysis, were reported in osmophore epidermal cells of several Orchidaceae species (Davies and Stpiczyńska, 2014; Stpiczyńska and Davies, 2016) and indicate the possible role of the epidermal cells in scent production. In addition, a layer of osmiophilic material lined the tonoplast inner surface, as occurs in the epidermal and parenchyma vacuole of *J. oxyphylla* nectarless flowers is also evidence of scent production.

The emission of volatile substances is of short duration and is associated with the fast utilization of large amounts of starch grains (Stern et al., 1987; Vogel, 1990; Pacini and Nepi, 2007; de Melo et al., 2010; Wiśniewska et al., 2018). In fact, essences were histochemically detected since the beginning of anthesis in nectarless flowers. In fact, prominent periplasmic space containing lipid droplets in regions underlying the outer tangential walls indicate secretory activity of epidermal cells associated with the accumulation and release of the volatile compounds. Likewise, the occurrence of large mitochondria and abundant SER located in the cortical cytoplasm, besides plastids featured by a reduced electron density and lipid inclusions, together with numerous lipid droplets in the cytosol and vacuoles of the subepidermal layers, are subcellular features commonly associated with synthesis of volatile compounds (Cheniclet and Carde, 1985; Figueiredo and Pais, 1994; Ascensão et al., 1997; Turner et al., 1999; Possobom et al., 2015; Stpiczyńska and Davies, 2016).

A noteworthy feature of the disks from nectarless flowers was the coexistence of plastids with different inclusions, only lipids or combinations of carbohydrate and phenolic substances. The increase in the amount of different materials (lipid–carbohydrate–phenolic) within the plastids throughout anthesis, as here observed, has been demonstrated in Orchidaceae osmophores (Wiśniewska et al., 2018 and references therein). The involvement of plastids in the synthesis of scent components, mainly terpenoids and phenolic compounds, has been broadly discussed (Pridgeon and Stern, 1985; Stern et al., 1987; Pais and Figueiredo, 1994; Kowalkowska et al., 2012; Stpiczyńska and Davies, 2016; Wiśniewska et al., 2018). Besides protection against herbivores, pathogens, and UV radiation (Brillouet et al., 2013), phenolic compounds are known to occur in floral scent (Jürgens and Dötterl, 2004; Jürgens et al., 2006; Steiner et al., 2011; Wiśniewska et al., 2018).

Our ultrastructural observations suggest that volatile compounds produced in plastids, cross the plastid envelope to the profiles of SER or migrate independently in the cytosol, and finally reach the plasmalemma and leave the protoplast by eccrine mechanism (Fahn, 1979). Volatile compounds participate in the attraction of mutualists or in the deterrence of antagonists (Harborne, 1997; Raguso, 2004; Guimarães et al., 2008). The most remarkable feature of the ground parenchyma cells in the disk of nectarless flowers after 72 h of anthesis was the presence of amyloplasts with prominent starch grains and dense phenolic inclusions, together with amyloplasts with signs of starch hydrolysis, and large vacuoles containing membrane debris, flocculent materials and phenolic compounds that were engulfed in the vacuoles, as discussed earlier. Therefore, we suggest that the occurrence of continuous storage and successive degradation of starch grains allowed continued production of volatiles throughout nectarless flowers’ disks lifespan.

The floral disk was supplied only by phloem, and it is probable that pre-nectar from the sieve tubes move away from an apoplastic route via intercellular spaces and from cell walls (e.g., Wist and Davis, 2006). Afterward, stored starch is progressively hydrolyzed and polysaccharides are transported from the amyloplasts to the vacuoles by vesicles. Accumulation of dense material in the periplasmic spaces, cell walls and intercellular spaces of nectary parenchyma tissue suggest that the apoplast system is involved in nectar transportation (Nepi, 2007) in *J. oxyphylla*.

The occurrence of modified stomata with associated secretions across the entire epidermis of the floral disk indicates the site of secretion release to the disk surface (Gaffal et al., 1998; Wist and Davis, 2006). In addition, secretion seems to also be released through microchannels in the cuticular layer, as cuticle channels may increase porosity and facilitate the passage of macromolecules (Rocha and Machado, 2009; de Melo et al., 2010; Stpiczyńska et al., 2010; Machado et al., 2017b).

In addition, a symplastic pathway of pre-nectar could also occur in the floral disk via plasmodesmata, which connects parenchyma and epidermal cells (Vassilyev, 2010). Moreover, the presence of well-developed Golgi bodies, numerous profiles of RER secretory vesicles originated from RER or Golgi bodies close to the plasmalemma that fuses with it, plasmalemma sinuous in outline, and developed periplasmic space, indicate vesicle-mediated process of secretion, providing evidence of a granulocrine mechanism of nectar release (Fahn, 1979). For lipophilic substances, as they are commonly present as droplets close to the plasma membrane or inside the periplasmic spaces, there is evidence of eccrine mechanism (Fahn, 1979), where the molecules cross the plasma membrane by active transport (Vassilyev, 2010). Both mechanisms of secretion release from the protoplast seem to occur simultaneously in the disk of nectariferous flowers, while in nectarless flowers, the eccrine mechanism is predominant.

Evidences of cell divisions in the nectary parenchyma, with the newly derivate cells integrating into the secretory tissue, were a common cytological feature to nectariferous and nectarless flowers. This process, quite similarly to that which occurs in meristematic tissues, has been reported in nectaries (Nepi, 2007) and secretory canals and cavities (Machado et al., 2017a) revealing the regenerative potential of secretory cells.

Although the occurrence of chloro-amyloplasts with undeveloped inner membranes in the subnectary parenchyma is an ultrastructural indication of the production of carbohydrates (Nepi, 2007), we have strong evidences that the carbohydrate supply comes largely from the hydrolysis of starch grains stored in amyloplasts. Diversity of plastids types, as here observed, including plastids with starch grains, undifferentiated plastid with osmiophilic bodies, chloro-amyloplasts, chloroplasts with poorly developed thylakoids or plastids with thylakoid-like membranes are common in floral nectaries of different taxa, mainly orchid species (Nepi, 2007). In a general way, undifferentiated plastids occur in the very early stages of nectary development, undergo some divisions before beginning to differentiate (Pacini et al., 1992; Nepi et al., 1996) and close to flower anthesis, chloro-amyloplasts are generally present in nectary parenchyma when secretion begins (Nepi, 2007). Contrary to previously investigated species, in the present study, undifferentiated plastids (or proplastids) and evidences of plastids division, were detected in nectary parenchyma cells at 48 h of anthesis, when nectar release stopped and amyloplasts were degenerated. A similar pattern of plastid differentiation was verified in nectarless flowers throughout anthesis. Based on this, we speculate that in *J. oxyphylla* there is no conversion of amyloplasts in elaioplasts, but differentiation of proplastids throughout the flowers lifespan.

### Chemical Composition of Floral Disks, of Secretion and Ecological Implications

The chemical analysis revealed that the nectarless disks do in fact produce volatile compounds. Generally, the disks might produce compounds that represent the floral scent as a whole or they might produce volatiles that add to an overall more complex scent (Dötterl and Jürgens, 2005; Dötterl and Vereecken, 2010). In present study, the compounds detected in the disk samples were also detected in floral headspace, and it remains to be tested how the disk contributes to the floral headspace and whether other flower organs also release these components. Nevertheless, detected compounds might have an impact on pollinator attraction in spite of nectarlessness. Indeed, all four compounds have been reported to be released by other bee-pollinated plants (e.g., Knudsen et al., 2006; Steiner et al., 2011) and phenylacetaldehyde is even a known attractant for bees of different families (Dötterl and Vereecken, 2010). This suggests that especially phenylacetaldehyde, but potentially also the other compounds, are involved in attracting the bee pollinators of *J. oxyphylla*. Bees are known to use floral volatiles to discriminate among resourceful and resourceless flowers of a given species (Dötterl and Vereecken, 2010). Thus, especially naïve bees might be cheated by the scent and visit nectarless flowers.

Even though plants with nectarless flowers are said to take advantage of higher fitness as they do not spend energy on nectar production (Thakar et al., 2003), there may be a counterbalance in this scenario, since pollinators may avoid visiting these flowers based on previous learning of nectar absence (Smithson and MacNair, 1997; Ferdy et al., 1998; Gumbert and Kunze, 2001; Vásquez and Barradas, 2018). In our focus species, approximately half of the flowers did not produce nectar, but produced volatile substances. These compounds could have a role in the maintenance of this plant–pollinator interaction, counterweighing the reduced attractiveness of resourceless flowers. So, *J. oxyphylla* might be taking a different path than Bignoniaceae species in which the complete loss of the ability to produce nectar was associated to disk loss (Umaña et al., 2011).

Additionally, *J. oxyphylla* presents a glandular and developed staminode, which is covered by abundant glandular trichomes secreting terpene and steroids that may participate in plant–pollinator interactions and be collected by Euglossini bees, such as *Eulaema nigrita* (Guimarães et al., 2008). The phenolic, lipophilic, and volatile compounds revealed by histochemistry in the secretory disk of both nectarless and nectariferous flowers are among most widely distributed compounds in angiosperms (Harborne, 1997). Phenolics may have various roles in plant–pollinator interactions being part of scent, taste and color (Harborne, 1985, 1997; Nishida, 2002). Additionally, lipophilic compounds present in the nectariferous disks may enrich nectar secretion with an additional energy source (Nicolson and Thornburg, 2007), which provides a long-term metabolic fuel for pollinators (Levin et al., 2017). The presence of lipids in nectar was also referred for other *Jacaranda* species (Baker and Baker, 1975; Kram et al., 2008).

In this study, we showcase the cellular basis of nectariferous flowers with different nectar production rhythms in the same plant species. Additionally, we bring for the first time a cellular characterization of nectarless flowers’ disks, which showed an unexpected production of lipophilic, phenolic and volatile substances. These changes in the functioning of the floral disk may influence plant–pollinator interaction. So, in the first and second days of anthesis, while nectariferous flowers release nectar in variable amounts and rhythms, nectarless flowers were involved in the secretion of other substances that could attract pollinators. These substances could either attract cheated nectarivores, by chemical signaling trough volatile compounds emission, or attract bees that are searching for floral resource.

## Future Directions And Conclusion

This study brings a broad panorama of nectarless flowers distribution and their cellular functional changes; however, we still have limitations to deeply interpret these findings in the light of plant–pollinator interactions. A question that arose from this study is if, like *Brassica rapa* (Gervasi and Schiestl, 2017), *J. oxyphylla* flowers present honest signals related to the presence of nectar, which could explain the low rates of pollinator visitation described by Guimarães et al. (2008). We should now evaluate if nectarless disks are contributing with exclusive compounds to floral scent, if the different types of flowers (early and late nectar producers, nectarless flowers) differ in their floral scents, if there is a temporal pattern of floral scent within a specific type throughout anthesis, and experimentally test the effect of the released volatile compounds on insect behavior. So, our next step is to evaluate these aspects in order to better understand the ecological implications of the cellular functional changes in secretory disks and of nectarlessness.

In conclusion, this study proposes a new paradigm, in which nectarlessness, instead of representing an energy saving strategy (Southwick, 1984; Pyke, 1991), could actually denote a higher energy investment, as the disks from nectarless flowers are producing volatile compounds instead of nectar. The little volume of nectar and the uncertainty that pollinator experience in finding a nectariferous flower, associated to the cellular functional changes in flowers’ disks, paint a picture of what could be a transition from a nectar-based pollination system to another resource-based or even to a deceit mechanism of pollination in *J. oxyphylla*.

## Author Contributions

EG and SM contributed to the conception and design of the study. EG, PT, and SM organized the data and wrote the first draft of the manuscript. EG and PT performed the statistical analysis. LDS, LAJ, and SD performed the chemical analysis. EG, PT, and SD interpreted the results of the chemical analysis. SM performed TEM analysis. All authors contributed to manuscript revision, read and approved the submitted version.

## Conflict of Interest Statement

The authors declare that the research was conducted in the absence of any commercial or financial relationships that could be construed as a potential conflict of interest.

## References

[B1] Adams R. P. (2007). *Identification of Essential Oil Components by Gas Chromatography/ Mass Spectrometry*, 4th Edn Carol Stream, IL: Allured Publishing Corporation.

[B2] Alcantara S. Lohmann L. G. (2010). Evolution of floral morphology and pollination system in Bignonieae (Bignoniaceae). *Am. J. Bot.* 97 782–796. 10.3732/ajb.0900182 21622444

[B3] Anand C. Umranikar C. Shintre P. Damle A. Kale J. Joshi J. (2007). Presence of two types of flowers with respect to nectar sugar in two gregariously flowering species. *J. Biosci.* 32 769–774. 10.1007/s12038-007-0077-1 17762150

[B4] Antoń S. Kamińska M. Stpiczyńska M. (2012). Comparative structure of the osmophores in the flowers of *Stanhopea graveolens* lindley and *Cycnoches chlorochilon* Klotzsch (Orchidaceae). *Acta Agrobot.* 65 11–22. 10.5586/aa.2012.054

[B5] Antoń S. Komoń-Janczara E. Denisow B. (2017). Floral nectary, nectar production dynamics and chemical composition in five nocturnal *Oenothera* species (Onagraceae) in relation to floral visitors. *Planta* 246 1051–1067. 10.1007/s00425-017-2748-y 28779217 PMC5653728

[B6] Armbruster W. S. Muchhala N. (2009). Associations between floral specialization and species diversity: cause, effect, or correlation? *Evol. Ecol.* 23 159–179. 10.1007/s10682-008-9259-z

[B7] Ascensão L. Marques N. Pais M. S. (1997). Peltate glandular trichomes of *Leonotis leonurus* leaves: ultrastructure and histochemical characterization of secretions. *Int. J. Plant Sci.* 158 249–258. 10.1086/297436

[B8] Baker H. G. Baker I. (1975). “Studies of nectar-constitution and pollinator plant coevolution,” in *Coevolution of Animals and Plants*, eds Gilbert L. E. Raven P. H. (New York, NY: Columbia University Press), 126–152.

[B9] Bell G. (1986). The evolution of empty flowers. *J. Theor. Biol.* 118 253–258. 10.1016/S0022-5193(86)80057-1

[B10] Brillouet J. M. Romieu C. Schoefs B. Solymosi K. Cheynier V. Fulcrand H. (2013). The tannosome is an organelle forming condensed tannins in the chlorophyllous organs of Tracheophyta. *Ann. Bot.* 112 1003–1014. 10.1093/aob/mct168 24026439 PMC3783233

[B11] Brink D. deWet J. M. (1980). Interpopulation variation in nectar production in *Aconitum columbianum* (Ranunculaceae). *Oecologia* 47 160–163. 10.1007/BF00346814 28309465

[B12] Cheniclet C. Carde J. P. (1985). Presence of leucoplasts in secretory cells and of monoterpenes in the essential oil: a correlative study. *Isr. J. Bot.* 34 219–238. 10.1080/0021213X.1985.10677023

[B13] Chittka L. Thomson J. D. (2001). *Cognitive Ecology of Pollination: Animal Behaviour and Floral Evolution.* Cambridge: Cambridge University Press 10.1017/CBO9780511542268

[B14] Corbet S. A. (2003). Nectar sugar content: estimating standing crop and secretion rate in the field. *Apidologie* 34 1–10. 10.1051/apido:2002049

[B15] Cruden R. W. Hermann S. M. Peterson S. (1983). “Patterns of nectar production and plant-pollinator coevolution,” in *The Biology of Nectarines*, eds Bentley B. Elias T. S. (New York, NY: Columbia University Press), 80–125.

[B16] Curry K. J. McDowell L. M. Judd W. S. Stern W. L. (1991). Osmophores, floral features, and systematics of *Stanhopea* (Orchidaceae). *Am. J. Bot.* 78 610–623. 10.2307/2445082 27798718

[B17] David R. Carde J. P. (1964). Coloration différentielle dês inclusions lipidique et terpeniques dês pseudophylles du Pin maritime au moyen du reactif Nadi. *C. R. Hebd. Séances Acad. Sci.* 258 1338–1340.

[B18] Davies K. L. Stpiczyńska M. (2014). Labellar anatomy and secretion in *Bulbophyllum* Thouars (Orchidaceae: Bulbophyllinae) sect. Racemosae Benth. and Hook. f. *Ann. Bot.* 114 889–911. 10.1093/aob/mcu153 25122654 PMC4171068

[B19] de Melo M. C. Borba E. L. Paiva E. A. S. (2010). Morphological and histological characterization of the osmophores and nectaries of four species of *Acianthera* (Orchidaceae: Pleurothallidinae). *Plant Syst. Evol.* 286 141–151. 10.1007/s00606-010-0294-1

[B20] Dötterl S. Jürgens A. (2005). Spatial fragrance patterns in flowers of *Silene latifolia*: lilac compounds as olfactory nectar guides? *Plant Syst. Evol.* 255 99–109. 10.1007/s00606-005-0344-2

[B21] Dötterl S. Vereecken N. J. (2010). The chemical ecology and evolution of bee–flower interactions: a review and perspectives. *Can. J. Zool.* 88 668–697. 10.1139/Z10-031

[B22] Dötterl S. Wolfe L. M. Jürgens A. (2005). Qualitative and quantitative analyses of flower scent in *Silene latifolia*. *Phytochemistry* 66 203–213. 10.1016/j.phytochem.2004.12.002 15652577

[B23] Durkee L. T. (1982). The floral and extra-floral nectaries of *Passiflora*. II. The extra-floral nectary. *Am. J. Bot.* 69 1420–1428. 10.2307/2443103 24349899

[B24] Durkee L. T. (1983). “The ultrastructure of floral and extrafloral nectaries,” in *The Biology of Nectaries*, eds Bentley B. Elias T. (New York, NY: Columbia University Press), 1–29.

[B25] Durkee L. T. Baird C. W. Cohen P. F. (1984). Light and electron microscopy of the resin glands of *Passiflora foetida* (Passifloraceae). *Am. J. Bot.* 71 596–602. 10.2307/2443335

[B26] Etl F. Berger A. Weber A. Schönenberger J. Dötterl S. (2016). Nocturnal plant bugs use cis-Jasmone to locate inflorescences of an Araceae as feeding and mating site. *J. Chem. Ecol.* 42 300–304. 10.1007/s10886-016-0688-9 27074793 PMC4867150

[B27] Fahn A. (1979). *Secretory Tissues in Plants.* New York, NY: Academic Press.

[B28] Fahn A. Shimony C. (2001). Nectary structure and ultrastructure of unisexual flowers of *Ecballium elaterium* (L.) A. Rich.(Cucurbitaceae) and their presumptive pollinators. *Ann. Bot.* 87 27–33. 10.1006/anbo.2000.1287

[B29] Feinsinger P. (1978). Ecological interactions between plants and hummingbirds in a successional tropical community. *Ecol. Monogr.* 48 269–287. 10.2307/2937231

[B30] Ferdy J. B. Gouyon P. H. Moret J. Godelle B. (1998). Pollinator behavior and deceptive pollination: learning process and floral evolution. *Am. Nat.* 152 696–705. 10.1086/286200 18811344

[B31] Figueiredo A. C. Pais M. S. S. (1994). Ultrastructural aspects of the glandular cells from the secretory trichomes and from the cell suspension cultures of *Achillea millefolium* L. ssp. *millefolium*. *Ann. Bot.* 74 179–190. 10.1006/anbo.1994.1107

[B32] Figueiredo A. C. S. Pais M. S. (1992). Ultrastructural aspects of the nectary spur of *Limodorum abortivum* (L) Sw.(Orchidaceae). *Ann. Bot.* 70 325–331. 10.1093/oxfordjournals.aob.a088481

[B33] Firmage D. H. Cole F. R. (1988). Reproductive success and inflorescence size of *Calopogon tuberosus* (Orchidaceae). *Am. J. Bot.* 75 1371–1377. 10.2307/2444460

[B34] Gaffal K. P. Heimler W. El-Gammal S. (1998). The floral nectary of Digitalis purpurea L., structure and nectar secretion. *Ann. Bot.* 81 251–262. 10.1006/anbo.1997.0546

[B35] Galetto L. (1995). Nectary structure and nectar characteristics in some Bignoniaceae. *Plant Syst. Evol.* 196 99–121. 10.1007/BF00985338

[B36] Galetto L. Bernardello G. (2005). “Rewards in flowers: nectar,” in *Practical Pollination Biology*, eds Dafni A. Kevan P. G. Husband B. C. (Cambridge: Enviroquest Ltd.), 261–313.

[B37] Gentry A. H. (1974). Coevolutionary patterns in central American Bignoniaceae. *Ann. Mo. Bot. Gard.* 61 728–759. 10.2307/2395026

[B38] Gentry A. H. Morawetz W. (1992). “Jacaranda,” in *Bignoniaceae - Part II (Tribe Tecomeae), Flora Neotropica: Monograph 25(2)*, ed. Gentry A. H. (New York, NY: Organization for Flora Neotropica), 51–105.

[B39] Gervasi D. D. Schiestl F. P. (2017). Real-time divergent evolution in plants driven by pollinators. *Nat. Commun.* 8:14691. 10.1038/ncomms14691 28291771 PMC5424062

[B40] Gigord L. D. Macnair M. R. Stritesky M. Smithson A. (2002). The potential for floral mimicry in rewardless orchids: an experimental study. *Proc. R. Soc. Lond. B Biol. Sci.* 269 1389–1395. 10.1098/rspb.2002.2018 12079663 PMC1691035

[B41] Gilbert F. S. Haines N. Dickson K. (1991). Empty flowers. *Funct. Ecol.* 5 29–39. 10.2307/2389553

[B42] Gleizes M. Carde J. P. Pauly G. Bernard-Dagan C. (1980). In vivo formation of sesquiterpene hydrocarbons in the endoplasmic reticulum of pine. *Plant Sci. Lett.* 20 79–90. 10.1016/0304-4211(80)90026-7

[B43] Guimarães E. Di Stasi L. C. Maimoni-Rodella R. D. C. S. (2008). Pollination biology of *Jacaranda oxyphylla* with an emphasis on staminode function. *Ann. Bot.* 102 699–711. 10.1093/aob/mcn152 18765441 PMC2712375

[B44] Guimarães E. Nogueira A. Machado S. R. (2016). Floral nectar production and nectary structure of a bee-pollinated shrub from Neotropical savanna. *Plant Biol.* 18 26–36. 10.1111/plb.12370 26194742

[B45] Gumbert A. Kunze J. (2001). Colour similarity to rewarding model plants affects pollination in a food deceptive orchid. *Orchis boryi*. *Biol. J. Linn. Soc.* 72 419–433. 10.1111/j.1095-8312.2001.tb01328.x

[B46] Harborne J. B. (1985). “Phenolics and plant defence”, in *Biochemistry of Plant Phenolics*, eds Van Sumere C. F. Lea P. J. (New York, NY: Oxford University Press), 393–408.

[B47] Harborne J. B. (1997). “Plant secondary metabolism,” in *Plant Ecology*, ed. Crawley M. J. (Berlin: Blackwell Publishing), 132–155.

[B48] Hauk W. D. (1997). A review of the genus *Cydista* (Bignoniaceae). *Ann. Mo. Bot. Gard.* 84 815–840. 10.2307/2992028

[B49] Heinrich B. (1975). Bee flowers: a hypothesis on flower variety and blooming times. *Evolution* 29 325–334. 10.1111/j.1558-5646.1975.tb00212.x 28555846

[B50] Hobbhahn N. Johnson S. D. Bytebier B. Yeung E. C. Harder L. D. (2013). The evolution of floral nectaries in Disa (Orchidaceae: Disinae): recapitulation or diversifying innovation? *Ann. Bot.* 112 1303–1319. 10.1093/aob/mct197 23997231 PMC3806529

[B51] Johansen D. A. (1940). *Plant Microtechnique.* New York, NY: McGraw.

[B52] Johnson S. D. (1994). Evidence for Batesian mimicry in a butterfly-pollinated orchid. *Biol. J. Linn. Soc.* 53 91–104. 10.1111/j.1095-8312.1994.tb01003.x

[B53] Johnson S. D. (2000). Batesian mimicry in the non-rewarding orchid *Disa pulchra*, and its consequences for pollinator behaviour. *Biol. J. Linn. Soc.* 71 119–132. 10.1111/j.1095-8312.2000.tb01246.x

[B54] Juillet N. Gonzalez M. A. Page P. A. Gigord L. D. B. (2007). Pollination of the European food-deceptive *Traunsteinera globosa* (Orchidaceae): the importance of nectar-producing neighbouring plants. *Plant Syst. Evol.* 265 123–129. 10.1007/s00606-006-0507-9

[B55] Jürgens A. Dötterl S. (2004). Chemical composition of anther volatiles in Ranunculaceae: genera-specific profiles in *Anemone*, Aquilegia, *Caltha*, *Pulsatilla*, Ranunculus, and *Trollius* species. *Am. J. Bot.* 91 1969–1980. 10.3732/ajb.91.12.1969 21652345

[B56] Jürgens A. Dötterl S. Meve U. (2006). The chemical nature of fetid floral odours in stapeliads (Apocynaceae-Asclepiadoideae-Ceropegieae). *New Phytol.* 172 452–468. 10.1111/j.1469-8137.2006.01845.x 17083676

[B57] Karnovsky M. J. (1965). A formaldehyde-glutaraldehyde fixative of high osmolarity for use in electron microscopy. *J. Cell Biol.* 27 137–138.

[B58] Knudsen J. T. Eriksson R. Gershenzon J. Ståhl B. (2006). Diversity and distribution of floral scent. *Bot. Rev.* 72 1–120. 10.1663/0006-8101200672

[B59] Kowalkowska A. K. Kozieradzka-Kiszkurno M. Turzyński S. (2015). Morphological, histological and ultrastructural features of osmophores and nectary of *Bulbophyllum* wendlandianum (Kraenzl.) Dammer (B. section *Cirrhopetalum* Lindl., Bulbophyllinae Schltr., Orchidaceae). *Plant Syst. Evol.* 301 609–622. 10.1007/s00606-014-1100-2

[B60] Kowalkowska A. K. Margońska H. B. Kozieradzka-Kiszkurno M. Bohdanowicz J. (2012). Studies on the ultrastructure of a three-spurred fumeauxiana form of *Anacamptis pyramidalis*. *Plant Syst. Evol.* 298 1025–1035. 10.1007/s00606-012-0611-y

[B61] Kram B. W. Bainbridge E. A. Perera M. A. Carter C. (2008). Identification, cloning and characterization of a GDSL lipase secreted into the nectar of *Jacaranda mimosifolia*. *Plant Mol. Biol.* 68 173–183. 10.1007/s11103-008-9361-1 18553138

[B62] Labandeira C. C. (2002). “The history of associations between plants and animals,” in *Plant Animal Interactions: an Evolutionary Approach*, eds Herrera C. M. Pellmyr O. (Hoboken, NJ: John Wiley and Sons), 26–76.

[B63] Levin E. McCue M. D. Davidowitz G. (2017). More than just sugar: allocation of nectar amino acids and fatty acids in a Lepidopteran. *Proc. R. Soc. Biol. Sci.* 284:20162126. 10.1098/rspb.2016.2126 28148746 PMC5310601

[B64] Lopes A. V. Vogel S. Machado I. C. (2002). Secretory trichomes, a substitutive floral nectar source in Lundia A. DC. (Bignoniaceae), a genus lacking a functional disc. *Ann. Bot.* 90 169–174. 10.1093/aob/mcf169 12197514 PMC4240414

[B65] Lu N. N. Li X. H. Li L. Zhao Z. G. (2015). Variation of nectar production in relation to plant characteristics in protandrous *Aconitum gymnandrum*. *J. Plant Ecol.* 8 122–129. 10.1093/jpe/rtv020

[B66] Machado S. R. Canaveze Y. Rodrigues T. M. (2017a). Structure and functioning of oil cavities in the shoot apex of Metrodorea nigra A. St.-Hil.(Rutaceae). *Protoplasma* 254 1661–1674. 10.1007/s00709-016-1056-x 27957603

[B67] Machado S. R. Souza C. V. Guimarães E. (2017b). A reduced, yet functional, nectary disk integrates a complex system of floral nectar secretion in the genus *Zeyheria* (Bignoniaceae). *Acta Bot. Br.* 31 344–357. 10.1590/0102-33062016abb0279

[B68] Machado S. R. Gregório E. A. Guimarães E. (2005). Ovary peltate trichomes of *Zeyheria montana* (Bignoniaceae): developmental ultrastructure and secretion in relation to function. *Ann. Bot.* 97 357–369. 10.1093/aob/mcj042 16371445 PMC2803635

[B69] Maués M. M. de Oliveira P. E. A. Kanashiro M. (2008). Pollination biology in *Jacaranda copaia* (Aubl.) D. Don. (Bignoniaceae) at the “floresta nacional do tapajós”, central amazon, brazil. *Braz. J. Bot.* 31 517–527. 10.1590/S0100-84042008000300015

[B70] Mitchell T. C. Dötterl S. Schaefer H. (2015). Hawk-moth pollination and elaborate petals in Cucurbitaceae: the case of the Caribbean endemic Linnaeosicyos amara. *Flora* 216 50–56. 10.1016/j.flora.2015.08.004

[B71] Morisita M. (1959). Measuring of the dispersion of individuals and analysis of the distributional patterns. *Mem. Fac. Sci. Kyushu Univ. E* 2 215–235.

[B72] Morisita M. (1962). Iσ-index, a measure of dispersion of individuals. *Res. Popul. Ecol.* 4 1–7. 10.1007/BF02533903

[B73] Nepi M. (2007). “Nectary structure and ultrastructure,” in *Nectaries and Nectar*, eds Nicolson S. W. Pacini E. Nepi M. (Dordrecht: Springer), 129–166. 10.1007/978-1-4020-5937-7_3

[B74] Nepi M. Ciampolini F. Pacini E. (1996). Development and ultrastructure of *Cucurbita pepo* nectaries of male flowers. *Ann. Bot.* 78 95–104. 10.1006/anbo.1996.0100

[B75] Nepi M. Stpiczyńska M. (2008). The complexity of nectar: secretion and resorption dynamically regulate nectar features. *Naturwissenschaften* 95 177–184. 10.1007/s00114-007-0307-2 17922103

[B76] Nicolson S. W. Thornburg R. W. (2007). “Nectar chemistry,” in *Nectaries and Néctar*, eds Nicolson S. W. Paccini E. Nepi M. (Dordrecht: Springer), 215–264. 10.1007/978-1-4020-5937-7_5

[B77] Nishida R. (2002). Sequestration of defensive substances from plants by Lepidoptera. *Annu. Rev. Entomol.* 47 57–92. 10.1146/annurev.ento.47.091201.145121 11729069

[B78] O’Brien T. P. Feder N. McCully M. E. (1964). Polychromatic staining of plant cell walls by toluidine blue O. *Protoplasma* 59 368–373. 10.1007/BF01248568

[B79] Pacini E. Nepi M. (2007). “Nectar production and presentation,” in *Nectaries and Nectar*, eds Nicolson S. W. Pacini E. Nepi M. (Dordrecht: Springer), 167–214. 10.1007/978-1-4020-5937-7_4

[B80] Pacini E. Taylor P. E. Singh M. B. Knox R. B. (1992). “Plastid developmental pathways in some angiosperm reproductive cells,” in *Angiosperm Pollen and Ovules*, ed. Mulcahy G. B. (New York, NY: Springer Verlag), 36–42. 10.1007/978-1-4612-2958-2_8

[B81] Pais M. S. Figueiredo A. C. S. (1994). Floral nectaries from *Limodorum abortivum* (L) Sw and *Epipactis atropurpurea* Rafin (Orchidaceae): ultrastructural changes in plastids during the secretory process. *Apidologie* 25 615–626. 10.1051/apido:19940612

[B82] Paiva E. A. S. Machado S. R. (2007). The floral nectary of *Hymenaea stigonocarpa* (Fabaceae, Caesalpinioideae): structural aspects during floral development. *Ann. Bot.* 101 125–133. 10.1093/aob/mcm268 17951584 PMC2701834

[B83] Peters G. (2017). *Userfriendlyscience: Quantitative Analysis Made Accessible., R Package Version 0.7.0.* Available at: http://userfriendlyscience.com

[B84] Possobom C. C. Machado S. R. (2018). Elaiophores in three Neotropical Malpighiaceae species: a comparative study. *Plant Syst. Evol.* 304 15–32. 10.1007/s00606-017-1443-6

[B85] Possobom C. C. F. Guimarães E. Machado S. R. (2010). Leaf glands act as nectaries in *Diplopterys pubipetala* (Malpighiaceae). *Plant Biol.* 12 863–870. 10.1111/j.1438-8677.2009.00304.x 21040301

[B86] Possobom C. C. F. Guimarães E. Machado S. R. (2015). Structure and secretion mechanisms of floral glands in *Diplopterys pubipetala* (Malpighiaceae), a neotropical species. *Flora* 211 26–39. 10.1016/j.flora.2015.01.002

[B87] Pridgeon A. M. Stern W. L. (1983). Ultrastructure of osmophores in *Restrepia* (Orchidaceae). *Am. J. Bot.* 70 1233–1243. 10.2307/2443293

[B88] Pridgeon A. M. Stern W. L. (1985). Osmophores of *Scaphosepalum* (Orchidaceae). *Bot. Gaz.* 146 115–123. 10.1086/337505

[B89] Pyke G. H. (1991). What does it cost a plant to produce floral nectar? *Nature* 350 58–59. 10.1038/350058a0

[B90] Pyke G. H. (2010). “Optimal foraging and plant–pollinator co-evolution,” in *Encyclopedia of Animal Behavior* Vol. 2 eds Payne A. Starks P. T. Liebert A. (Cambridge, MA: Academic Press).

[B91] Pyke G. H. (2016). Floral nectar: pollinator attraction or manipulation? *Trends Ecol. Evol.* 31 339–341. 10.1016/j.tree.2016.02.013 26987770

[B92] Quinalha M. M. Nogueira A. Ferreira G. Guimarães E. (2017). Effect of mutualistic and antagonistic bees on floral resources and pollination of a savanna shrub. *Flora* 232 30–38. 10.1016/j.flora.2016.08.005

[B93] R Development Core Team. (2016). *R: A Language and Environment for Statistical Computing.* Vienna: R Foundation for Statistical Computing.

[B94] R Development Core Team. (2018). *R: A Language and Environment for Statistical Computing.* Vienna: R Foundation for Statistical Computing.

[B95] Raguso R. A. (2004). Why are some floral nectars scented? *Ecology* 85 1486–1494. 10.1890/03-0410

[B96] Ramalingam K. Ravindranath M. H. (1970). Histochemical significance of green metachromasia to toluidine blue. *Histochemie* 24 322–327. 10.1007/BF00278217 4100072

[B97] Real L. A. (1981). Nectar availability and bee-foraging on *Ipomoea* (Convolvulaceae). *Biotropica* 13 64–69. 10.2307/2388072

[B98] Reynolds E. S. (1963). The use of lead citrate at high pH as an electron-opaque stain in electron microscopy. *J. Cell Biol.* 17 208. 10.1083/jcb.17.1.208 13986422 PMC2106263

[B99] Rivera G. L. (2000). Nuptial nectary structure of Bignoniaceae from Argentina. *Darwiniana* 38 227–239.

[B100] Rocha J. F. Machado S. R. (2009). Anatomy, ultrastructure and secretion of *Hibiscus pernambucensis* Arruda (Malvaceae) extrafloral nectary. *Braz. J. Bot.* 32 489–498. 10.1590/S0100-84042009000300008

[B101] Schaefer H. M. Ruxton G. D. (2011). *Plant-Animal Communication.* Oxford: OUP 10.1093/acprof:osobl/9780199563609.001.0001 21902751

[B102] Scrucca L. (2011). Model-based {SIR} for dimension reduction. *Comput. Stat. Data Anal.* 5 3010–3026. 10.1016/j.csda.2011.05.006

[B103] Smithson A. Gigord L. D. (2001). Are there fitness advantages in being a rewardless orchid? Reward supplementation experiments with Barley robertiana. *Proc. R. Soc. Lond. B Biol. Sci.* 268 1435–1441. 10.1098/rspb.2001.1705 11454285 PMC1088760

[B104] Smithson A. MacNair M. R. (1997). Negative frequency-dependent selection by pollinators on artificial flowers without rewards. *Evolution* 51 715–723. 10.1111/j.1558-5646.1997.tb03655.x 28568581

[B105] Southwick E. E. (1984). Photosynthate allocation to floral nectar: a neglected energy investment. *Ecology* 65 1775–1779. 10.2307/1937773

[B106] Souza C. V. Nepi M. Machado S. R. Guimaraes E. (2017). Floral biology, nectar secretion pattern and fruit set of a threatened Bignoniaceae tree from Brazilian tropical forest. *Flora* 227 46–55. 10.1016/j.flora.2016.12.007

[B107] Steiner K. E. Kaiser R. Dötterl S. (2011). Strong phylogenetic effects on floral scent variation of oil-secreting orchids in South Africa. *Am. J. Bot.* 98 1663–1679. 10.3732/ajb.1100141 21965135

[B108] Stern W. L. Curry K. J. Pridgeon A. M. (1987). Osmophores of *Stanhopea* (Orchidaceae). *Am. J. Bot.* 74 1323–1331. 10.2307/2444310

[B109] Stpiczyńska M. (2003). Nectar resorption in the spur of *Platanthera chlorantha* Custer (Rchb.) Orchidaceae–structural and microautoradiographic study. *Plant Syst. Evol.* 238 119–126. 10.1007/s00606-002-0281-2

[B110] Stpiczyńska M. Davies K. L. (2016). Evidence for the dual role of floral secretory cells in *Bulbophyllum*. *Acta Biol. Crac. Ser. Bot.* 58 57–69. 10.1515/abcsb-2016-0013

[B111] Stpiczyńska M. Davies K. L. Kamińska M. (2010). Comparative anatomy of the nectary spur in selected species of Aeridinae (Orchidaceae). *Ann. Bot.* 107 327–345. 10.1093/aob/mcq246 21183455 PMC3043926

[B112] Stpiczyńska M. Milanesi C. Faleri C. Cresti M. (2005). Ultrastructure of the nectary spur of Platanthera chlorantha (Custer) Rchb.(*Orchidaceae*) during successive stages of nectar secretion. *Acta Biol. Crac. Ser. Bot.* 47 111–119.

[B113] Subramanian R. B. Arumugasamy K. Inamdar J. A. (1990). Studies in the secretory glands of *Hiptage sericea* (Malpighiaceae). *Nord. J. Bot.* 10 57–62. 10.1111/j.1756-1051.1990.tb01753.x

[B114] Thakar J. D. Kunte K. Chauhan A. K. Watve A. V. Watve M. G. (2003). Nectarless flowers: ecological correlates and evolutionary stability. *Oecologia* 136 565–570. 10.1007/s00442-003-1304-6 12838401

[B115] Tölke E. E. A. D. Galetto L. Machado S. R. Lacchia A. P. S. Carmello-Guerreiro S. M. (2015). Stages of development of the floral secretory disk in *Tapirira guianensis* Aubl.(Anacardiaceae), a dioecious species. *Bot. J. Linn. Soc.* 179 533–544. 10.1111/boj.12340

[B116] Torres C. Galetto L. (1998). Patterns and implications of floral nectar secretion, chemical composition, removal effects and standing crop in *Mandevilla pentlandiana* (Apocynaceae). *Bot. J. Linn. Soc.* 127 207–223. 10.1111/j.1095-8339.1998.tb02098.x

[B117] Turner G. Gershenzon J. Nielson E. E. Froehlich J. E. Croteau R. (1999). Limonene synthase, the enzyme responsible for monoterpene biosynthesis in peppermint, is localized to leucoplasts of oil gland secretory cells. *Plant Physiol.* 120 879–886. 10.1104/pp.120.3.879 10398724 PMC59327

[B118] Umaña M. N. Stevenson P. R. Alcantara S. Lohmann L. G. (2011). Pollination in the deceptive species *Bignonia corymbosa* (Bignoniaceae): a plant who deceives their floral visitors. *Int. J. Plant Reprod. Biol.* 3 15–22.

[B119] Vásquez V. Barradas I. (2018). A plant–pollinator system: how learning versus cost-benefit can induce periodic oscillations. *Int. J. Biomath.* 11:1850024. 10.1142/S1793524518500249 30114894

[B120] Vassilyev A. E. (2010). On the mechanisms of nectar secretion: revisited. *Ann. Bot.* 105 349–354. 10.1093/aob/mcp302 20053630 PMC2826252

[B121] Vogel S. (1990). *The Role of Scent Glands in Pollination: on the Structure and Function of Osmophores.* New Delhi: Amerind.

[B122] Wickham H. (2009). *Ggplot2: Elegant Graphics for Data Analysis.* New York, NY: Springer-Verlag 10.1007/978-0-387-98141-3

[B123] Willmer P. (2011). *Pollination and Floral Ecology.* Princeton: Princeton University Press 10.1515/9781400838943

[B124] Wiśniewska N. Kowalkowska A. K. Kozieradzka-Kiszkurno M. Krawczyńska A. T. Bohdanowicz J. (2018). Floral features of two species of *Bulbophyllum* section Lepidorhiza Schltr.: B. levanae Ames and B. nymphopolitanum Kraenzl.(Bulbophyllinae Schltr. Orchidaceae). *Protoplasma* 255 485–499. 10.1007/s00709-017-1156-2 28913668

[B125] Wist T. J. Davis A. R. (2006). Floral nectar production and nectary anatomy and ultrastructure of *Echinacea purpurea* (Asteraceae). *Ann. Bot.* 97 177–193. 10.1093/aob/mcj027 16339769 PMC2803364

[B126] Zhao Z. Lu N. Conner J. K. (2016). Adaptive pattern of nectar volume within inflorescences: bumblebee foraging behavior and pollinator-mediated natural selection. *Sci. Rep.* 6:34499. 10.1038/srep34499 27687244 PMC5043277

[B127] Zimmerman M. (1981). Patchiness in the dispersion of nectar resources: probable causes. *Oecologia* 49 154–157. 10.1007/BF00349182 28309303

